# Codon usage and protein length-dependent feedback from translation elongation regulates translation initiation and elongation speed

**DOI:** 10.1093/nar/gkab729

**Published:** 2021-08-20

**Authors:** Xueliang Lyu, Qian Yang, Fangzhou Zhao, Yi Liu

**Affiliations:** Department of Physiology, The University of Texas Southwestern Medical Center, 5323 Harry Hines Blvd., Dallas, TX 75390, USA; State Key Laboratory of Agricultural Microbiology, College of Plant Science and Technology, Huazhong Agricultural University, Wuhan, Hubei 430070, China; Department of Physiology, The University of Texas Southwestern Medical Center, 5323 Harry Hines Blvd., Dallas, TX 75390, USA; Department of Physiology, The University of Texas Southwestern Medical Center, 5323 Harry Hines Blvd., Dallas, TX 75390, USA; Department of Physiology, The University of Texas Southwestern Medical Center, 5323 Harry Hines Blvd., Dallas, TX 75390, USA

## Abstract

Essential cellular functions require efficient production of many large proteins but synthesis of large proteins encounters many obstacles in cells. Translational control is mostly known to be regulated at the initiation step. Whether translation elongation process can feedback to regulate initiation efficiency is unclear. Codon usage bias, a universal feature of all genomes, plays an important role in determining gene expression levels. Here, we discovered that there is a conserved but codon usage-dependent genome-wide negative correlation between protein abundance and CDS length. The codon usage effects on protein expression and ribosome flux on mRNAs are influenced by CDS length; optimal codon usage preferentially promotes production of large proteins. Translation of mRNAs with long CDS and non-optimal codon usage preferentially induces phosphorylation of initiation factor eIF2α, which inhibits translation initiation efficiency. Deletion of the eIF2α kinase CPC-3 (GCN2 homolog) in *Neurospora* preferentially up-regulates large proteins encoded by non-optimal codons. Surprisingly, CPC-3 also inhibits translation elongation rate in a codon usage and CDS length-dependent manner, resulting in slow elongation rates for long CDS mRNAs. Together, these results revealed a codon usage and CDS length-dependent feedback mechanism from translation elongation to regulate both translation initiation and elongation kinetics.

## Graphical Abstract

**Graphical Abstract:**
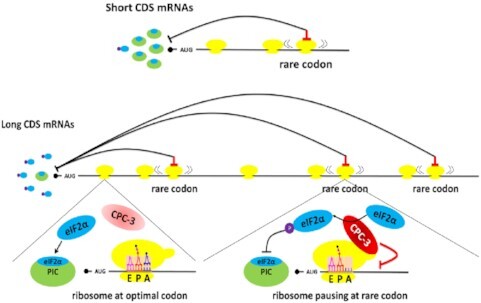
The mechanism of translation elongation feeding back to regulate translation initiation and elongation speed in a codon usage and CDS length-dependent manner. Rare codons cause ribosome pausing during elongation, resulting in CPC-3 activation and phosphorylation of eIF2a in a codon usage-and CDS length-dependent manner. Phosphorylated eIF2a inhibits translation initiation. In addition, CPC-3 also regulates translation elongation speed in a codon usage and CDS length-dependent manner.

## INTRODUCTION

Degeneracy of genetic codons allows 18 of 20 standard amino acids to be encoded by two to six synonymous codons. The preference for specific synonymous codons is a universal feature of all genomes (1–3). Although synonymous codons encode the same amino acid and synonymous mutations are often referred to as ‘silent’, genetic, biochemical, molecular, and bioinformatics studies have demonstrated that codon usage can influence gene expression levels and protein structures (3–9). Preferred codons (also called optimal codons) are enriched in genes that encode highly expressed proteins, and codon optimization can strongly increase endogenous and heterologous gene expression in diverse eukaryotes and prokaryotes (10–20). Codon usage regulates the speed of translation elongation during mRNA translation: Preferred codons speed up elongation, whereas rare codons slow down elongation and can cause ribosome pausing (7,21–24). The role of codon usage in translation kinetics regulates translation efficiency in part because rare codons can slow elongation and potentially cause premature translation termination (23). Codon usage can also impact mRNA stability and translation fidelity (5,25–28). In addition, the codon usage-dependent translation kinetics also regulates co-translational protein folding and protein functions in prokaryotic and eukaryotic organisms (6,16,21,29–32). Furthermore, there are translation-independent effects of codon usage due to the roles of nucleotide sequence in transcription, chromatin structure, splicing, mRNA structure, transcriptional termination and mRNA localization (7,13,28,33,34).

Synthesis of large proteins encounters many obstacles in cells. Errors in transcriptional, posttranscriptional and translational processes are expected to increase as coding sequence (CDS) length increases. Long mRNAs and large proteins are more likely to be degraded or damaged than shorter ones (35). In addition, large proteins have more protein domains, resulting in increased complexity in the protein folding process and greater likelihood of misfolding than small proteins. Previous studies have shown that CDS length negatively correlates with protein abundance, translation initiation rate, and ribosome density (22,36–40), suggesting the existence of mechanisms that preferentially inhibit translation of large proteins. Modeling studies suggest that the negative influence of CDS length on translation is likely due to less efficient ribosome recycling on mRNAs with longer compared to shorter CDS regions (39,41). Despite these issues, many large proteins are critical for cellular functions in diverse biological processes. The mechanisms that allow efficient production and proper folding of large proteins are not clear.

The best characterized mechanism that regulates translation efficiency is the initiation process, which largely determines the number of protein molecules that can be made from an individual mRNA transcript (42–44). Ribosome queuing near AUG start codon caused by ribosome stalling or collision impacts translation initiation efficiency (45,46). A previous study using a reporter gene in *Saccharomyces cerevisiae* suggested that rare codons near the start codon could inhibit translation initiation probably due to ribosome queuing near the start codon, whereas optimal codons near the start codon presumably result in rapid liberation of the start codon and therefore high translation initiation rates (47). However, other studies showed that codon usage near the start codon appears to influence translation initiation rate due to its effects on mRNA structures rather than translation elongation (48,49). Thus, the mechanism underlying the coordination between translation initiation and elongation under nutrient replete growth conditions and the role of codon usage in this process are still unclear.

In eukaryotes, translation initiation begins with the binding of the ternary complex (the aminoacylated initiator methionyl-tRNA (Met-tRNA_i_), GTP and the initiation factor 2 (eIF2)) to the 40S ribosome to form the pre-initiation complex (42–44,50). Phosphorylation of eIF2α, a subunit of eIF2, at serine 51 is an important regulator of translation initiation and is known to be induced by many types of stress conditions to result in global inhibition of translation initiation. The phosphorylation of eIF2α-GDP inhibits the guanine nucleotide exchange activity of eIF2B and blocks the recycling of unphosphorylated eIF2α-GDP into the translationally active form eIF2α-GTP. In higher eukaryotes, GCN2 is one of the several kinases responsible for the phosphorylation of eIF2α at serine 51 after its activation from an autoinhibited state (42,50,51). In fungi, however, the GCN2 homolog is the major and the only known eIF2α kinase responsible for eIF2α phosphorylation at serine 51 (52). Upon nutrient limitation or amino acid starvation or other stress conditions, GCN2 is activated and phosphorylates eIF2α to initiate the adaptive pro-survival integrated stress response, resulting in temporary translation repression of most mRNAs and activation of amino acid biosynthesis (53–56). In addition, mutation or depletion of enzymes required for tRNA modification can also trigger eIF2α phosphorylation (57,58). Although GCN2 can be activated by interacting with uncharged tRNA caused by amino acid starvation, recent evidence also suggests that GCN2 can also be activated by other mechanisms such as by interacting with the ribosomal P-stalk (59–61). Although the stress-induced eIF2α phosphorylation is expected to cause global translation repression, recent studies showed that this is not the case for low levels of eIF2α phosphorylation, suggesting that, under certain conditions, eIF2α phosphorylation has specific rather than a broad inhibitory effect on general translation (50,62).

The filamentous fungus *Neurospora crassa* exhibits a strong codon usage bias for C/G at wobble positions and has been an important experimental model system for studying the functions of codon usage biases (6,7,16). We have previously shown that codon usage plays an important role in regulating elongation speed and the co-translational protein folding process in *Neurospora* (9,16,21,63). Use of preferred codons speeds up the local rate of translation elongation while rare codons slow down translation elongation and potentially result in ribosome pausing and premature termination, a mechanism that can affect translation efficiency (21,23). We also showed that codon usage could influence gene expression levels by affecting transcription efficiency (13,33,64). These results led us to propose that codon usage represents a code within the genetic codons that regulates both gene expression level and protein structure.

In this study, we used *N. crassa* as a model system to understand the CDS length-dependent mechanism that regulates translation. We discovered that there is a conserved codon usage-dependent genome-wide negative correlation between protein abundance and length, suggesting that optimal codon usage is a mechanism that allows for efficient production of large proteins critical for cell functions. We found that translation of mRNAs with non-optimal codon usage preferentially induced eIF2α phosphorylation and reduced protein levels in a CDS length-dependent manner, indicating a feedback mechanism from translation elongation to control translation initiation. Furthermore, we showed that the GCN2 homolog, CPC-3, which is the only known kinase responsible for eIF2α phosphorylation in *Neurospora* (52), also regulates translation elongation rate in both codon usage and CDS length-dependent manners, resulting in slow elongation rates for mRNAs with long CDS regions. Together, these results revealed a codon usage- and CDS length-based feedback mechanism from translation elongation to regulate both translation initiation and elongation kinetics.

## MATERIALS AND METHODS

### *Neurospora* strains and growth conditions

The *N. crassa* wild-type (WT) strain FGSC 4200 (a) and the Δ*cpc-3* strain (65) were used in this study. Strains were cultured on slants containing 1 × Vogel's, 3% sucrose, and 1.5% agar. Liquid cultures were grown in 2% glucose medium (1 × Vogel's, 2% glucose). Specifically, fresh conidia (7–10 days post inoculation on slants) of the WT or Δ*cpc-3* strains were cultured in 50 ml 2% glucose medium in petri dishes at room temperature for 2 days. The cultures were cut into small discs with a diameter of 1 cm, and then the discs were transferred into flasks with the same liquid medium and were grown with orbital shaking (200 rpm) for 12 h before various experiments. Race tube medium contained 1 × Vogel's, 0.1% glucose, 0.17% arginine, and 1.5% agar. All the strains were cultured under constant light at room temperature unless otherwise specified. For chemical treated experiments, various indicated drugs were added into 2% glucose medium for the WT strain for 20 min before harvesting. 3-aminotriazole (3-AT, Sigma-Aldrich, Cat. No. 8056), puromycin (Puro, Sigma-Aldrich, Cat. No. 540411) and tigecycline (TIG, Sigma-Aldrich, Cat. No. PZ0021) were used at the final concentration of 50 mM, 0.06 mg/mL and 0.5 mg/mL, respectively. Anisomycin (ANS, Sigma-Aldrich, Cat. No. A9789) and cycloheximide (CHX, Sigma-Aldrich, Cat. No. C1988) were used at the indicated concentration.

### Plasmid construction, transformation and Δ*cpc-3* complementation

For gene expression at the *csr-1* locus in *N. crassa*, a basta-resistance (*bar)* gene was inserted downstream of the *ccg-1* promoter (Pccg-1) of a parental plasmid, Pcsr1, to create the Pcsr1-bar plasmid. Pcsr1-bar is a *csr-1*-targeting expression vector with an expression cassette in which Pccg-1 and *bar* flank the gene of interest (*edp* or *cpc-3* in this study), and this cassette is flanked by *csr-1* downstream and upstream fragments which serve as the recombination sites for double homologous recombination (66). Afterwards, the resulting plasmid was transformed into *N. crassa* strains by electroporation, transformants were screened for resistance to both glufosinate-ammonium (0.25 mg/ml, Sigma-Aldrich, Cat. No. 45520) and to cyclosporin A (5 μg/ml, Sigma-Aldrich, Cat. No. 30024), which resulted in >90% positive transformants. Homokaryatic strains were obtained by microconidia purification. To generate the Δ*cpc-3* complementation strains, a construct expressing the WT *cpc-3* (cloned from genomic DNA) with 3× Flag epitope tag under the control of the *ccg-1* promoter was introduced at the *csr-1* locus in the Δ*cpc-3* strain. The expression of the Flag-tagged CPC-3 and the rescue of eIF2α phosphorylation in the complementation strains were confirmed by immunoblotting (Supplementary Figure S1).

### RNA isolation and quantitative reverse transcription PCR (qRT-PCR)

The culture conditions were the same as described above. RNA extraction and qRT-PCR were performed as previously described (58). β-tubulin transcript (*NCU04054*) was quantified as an internal control. The primer pairs 5′-ACAACCCCTCACATCAACCAA-3′, 5′-CCGCCCTTGTCATCGTCATCC-3′ and 5′-GCGTATCGGCGAGCAGTT-3′, 5′-CCTCACCAGTGTACCAATGCA-3′ were used to amplify the reporter gene *edp* and β-tubulin gene, respectively. The primers for different versions of *edp* were designed to amplify the 5′ end region of the transcripts (5′UTR, 3× Flag and 8xGly linker), which is common to all the transgenes to ensure the same amplification efficiency.

### *In vitro* translation and protein analyses

*In vitro* translation assay was performed as previously described (21). Specifically, the *N. crassa* cell free lysate was obtained as previously described (21,67), except that the protease inhibitor cocktail from MedChemExpress company (Cat. No. HY-K0010) was used. Equal moles (0.65 pmol for each reaction) of different versions of mRNAs were individually added into *N. crassa* cell free lysate to translate for 15 min at 26°C unless otherwise specified. SDS-PAGE loading buffer was added into the samples immediately and followed by heating the samples at 98°C for 10 min.

Protein extraction was performed as previously described (58). For western blot analysis of the Flag-tagged EDP, the anti-Flag (Sigma-Aldrich, Cat. No. F3165) antibody was used. Densitometric analyses of the western blot results were performed using Image J.

For phosphatase treatment, total proteins from the WT and Δ*cpc-3* strains were obtained by using protein lysate buffer with or without phosphatase inhibitors (PP inhibitors: 25 mM NaF, 10 mM Na_4_P_2_O_7_.10H_2_O, 2 mM Na_3_VO_4_ and 1 mM EDTA). The protein extracts from lysate buffer without PP inhibitors were further treated with Lambda protein phosphatase (Lambda PP, NEB, Cat NO.: P0753S) according to its protocol.

### Cell-free translation assay to determine TFAs

To prepare the mRNA templates for *in vitro* translation, *in vitro* transcription of different mRNAs was performed as previously described (21). *In vitro* translation assays to determine TFAs were performed as previously described (21) and the luminescence of luciferase signal was recorded continuously in 20-s intervals.

### Ribosome profiling and mRNA-seq

The WT and Δ*cpc-3* strains expressing 1× WT-EDP, 3× WT-EDP, 1× OPT-EDP and 3× OPT-EDP, respectively, were used for ribosome profiling and accompanying mRNA-seq experiments under nutrient replete condition at room temperature (1 × Vogel's, 2% glucose). At least three biological replicates for the ribosome profiling experiment for each strain were used. Ribosome profiling experiments were performed as previously described (58). CHX was not added into cultures before sample collection and was only added into the lysate buffer at final concentration of 0.1 mg/ml. The ribosome profiling and mRNA-seq methods were described in the protocol for ARTseq Ribosome Profiling Kit (Yeast) (Illumina, Cat NO.: RPYSC12116). The adaptor in the kit was replaced with synthesized 5′-/5rApp/NNNNAGATCGGAAGAGCACACGTCT/3ddC/ to avoid potential adaptor ligation biases. The RT-primer in the kit was replaced with synthesized 5′-/5Phos/RNCGTCGGACTGTAGAACTCTG/iSp18/AGACGTGTGCTCTTCCGATCT to avoid potential ligation biases during the circularization process. The resulting libraries were sequenced by the BGI DNBseq platform.

### Bioinformatics analyses of ribosome profiling results

The workflow for ribosome profiling experiment data analysis is the same as previously described (58), with the following modifications: Both of the ribo-seq and RNA-seq reads were mapped to CDS regions of genes. Both ribosome protected fragment (RPF) and mRNA level for each gene were measured by Transcripts Per Kilobase Million (TPM). Ribosome density was measured by TPM of RPFs normalized by TPM of mRNAs. The calculation of the relative codon decoding time (RCDT) is the same as previously described (21,23). The raw reads of biological replicates of each strain were merged when we analyzed the ribosome profiling data. Ribo-seq and RNA-seq data from WT and Δ*cpc-3* strains expressing 1× WT-EDP, 3× WT-EDP, 1× OPT-EDP and 3× OPT-EDP, respectively, were regarded as four independent biological replicates when we performed genome-wide analyses for the WT and Δ*cpc-3* strains.

### Metabolic labeling

Fresh *Neurospora* conidia of the WT and Δ*cpc-3* strains were cultured separately in 50 ml 2% sucrose medium (1 × Vogel's, 2% sucrose) in flasks with orbital shaking (200 rpm). After culturing at 30°C for 8 h, EasyTag l-[^35^*S*]-methionine (PerkinElmer) was added into the medium for 45 min before sample collection. The same amounts of protein extracts (100 μg) from each sample were used to determine the levels of ^35^S incorporation as previously described (68).

### Polysome profiling

The culture condition for polysome profiling is the same as the metabolic labeling experiment. Cultures of the WT and Δ*cpc-3* strains were frozen in liquid nitrogen immediately after collection. Tissue samples were grounded into powder in liquid nitrogen and equal volume of the tissue powder of each sample was added the same volume of lysis buffer (1 × polysome buffer in ARTseq Ribosome Profiling Kit (Illumina, Cat. No. RPYSC12116), 1% Triton X-100, 0.1 mg/ml CHX, 1× protease inhibitor cocktail (EDTA-free, MedChemExpress, Cat. No. HY-K0010), 0.2 U/μl SUPERase•In (Invitrogen, Cat. No. AM2694) and 2 mM DTT). The lysates were then centrifugated at 15 000 rpm for 10 min before the *A*_260_ of the supernatant was measured by NanoDrop Microvolume Spectrophotometer. The *A*_260_/ml of the lysate was calculated according to the protocol for the ARTseq Ribosome Profiling Kit. The same OD amount (20 OD_260nm_) of the lysate for each sample was loaded onto 10–50% sucrose gradient buffer containing 20 mM HEPES (pH 7.6), 0.1 M KCl, 5 mM MgCl_2_, 10 μg/ml CHX, the 1× protease inhibitor cocktail and 10 units/ml SUPERase•In. The sucrose gradients were then centrifuged at 35 000 rpm for 2 h at 4°C using a SW41Ti rotor in a Beckman Coulter (Optima L-80 ultra) centrifuge. Sucrose gradients were analyzed using a BioLogic LP chromatography System (Bio-Rad, Cat. No. 731-8350).

### Mass spectrometry analyses

Cell culturing was performed as described in the metabolic labeling experiment above. For mass spectrometry (MS) analysis to compare the relative amounts of different proteins within the same sample, ∼100 μg proteins for each sample from the WT strain were run 1 cm into 7.5% SDS-PAGE gel. Gel slices were cut into small pieces for quantitative MS analysis. MaxQuant was used to analysis the MS data (69), and an intensity-based absolute quantification (iBAQ) value was used as a measure of protein abundance (70–72). For the quantitative MS analysis to compare the protein levels between the Δ*cpc-3* strain and its complementation strain, the cultures were grounded into powder in liquid nitrogen before adding equal volume of lysis buffer (50 mM triethylammonium bicarbonate (TEAB) and 5% SDS) to equal volume of the powder for each sample. The protein extracts were centrifuged at 12 000 rpm for 10 min and the supernatants were used for the subsequent TMT Mass Tagging and MS analyses. The protein concentration for each sample was measured and equal amounts of total proteins were used during the experiment. There are four repeats for each strain. The abundance data of the quantitative MS (TMT labeling) analysis were not normalized by molecular weights. The protein levels were determined by the abundance data normalized by the sum of all the raw abundance data in each sample. The *P*-values for the four replicates were determined by Student's two-tailed *t*-test, which were further adjusted by False Discovery Rate (FDR). Those with FDR values <0.05 were identified as differentially expressed proteins, either significantly up-regulated or down-regulated. All the MS analyses were performed by the UT Southwestern Proteomics facility.

### Codon manipulation, indices calculation and data collection from databases

The codon usage of luciferase and *edp* genes were optimized based on the *N*. *crassa* codon usage frequency from the Codon Usage Database (https://www.kazusa.or.jp/codon/). The WT and optimized (OPT) versions of luciferase genes are the same as previously described (21). The sequences of WT/OPT-*Luc*, WT/OPT-*edp*, and WT/OPT-*GFP* were shown in Supplementary Figure S2. The CBIs and tAIs were calculated using CodonW (http://codonw.sourceforge.net/) and stAIcalc (73), respectively. The tRNA copy number-related data used for calculating tAIs was collected from GtRNAdb database (http://gtrnadb.ucsc.edu/GtRNAdb2/). For the protein abundance data, the emPAI data of *N. crassa* was obtained previously in our lab (13) and the iBAQ data is obtained from this study. The publicly available protein abundance data of *S. cerevisiae*, *Drosophila melanogaster*, *Caenorhabditis elegans* and *Mus musculus* were used in the analyses (74–77).

### Gene functional enrichment analysis

The functional category (including Gene Ontology (GO), Interpro, and KEGG terms) enrichment analyses were performed with the functional annotation tool of the DAVID bioinformatics web server (http://david.abcc.ncifcrf.gov/), and the whole genome annotation was used as background. The genes of each enriched functional category, the enrichment fold change, and the various statistical parameters of the enrichment analysis including *P*-values, Bonferroni-corrected *P*-values, Benjamini-corrected *P*-values, and FDR values were determined.

## RESULTS

### Optimal codon usage has a conserved role in promoting the production of large proteins

**Figure 1.**
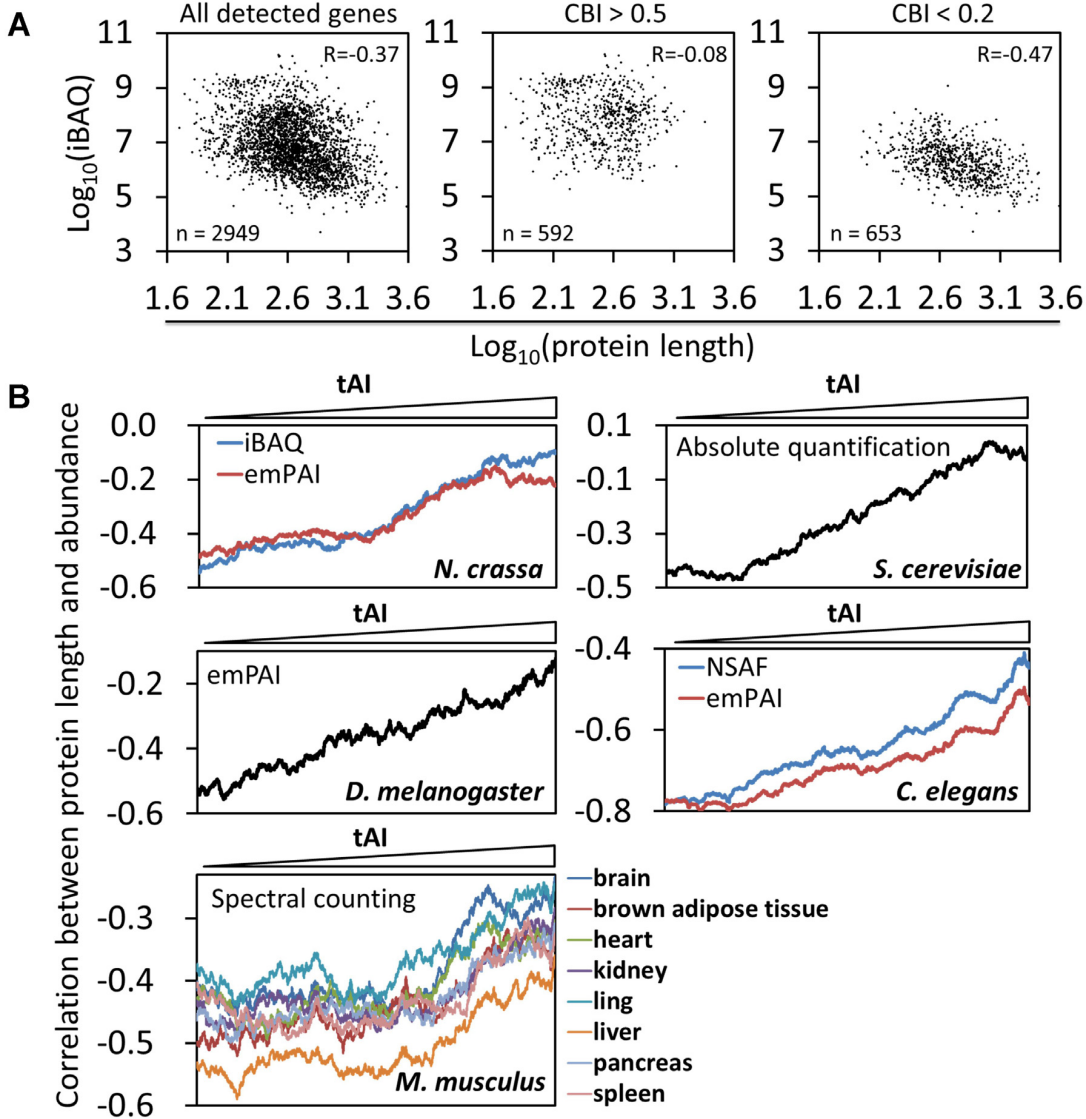
The negative correlation between protein length and abundance is codon usage-dependent and conserved in eukaryotes. (**A**) Scatter plots showing the correlations (Pearson's correlation coefficients) between protein length and abundance for *Neuropsora* genes genome-wide (left panel) and for genes with high CBIs (middle panel) and low CBIs (right panel) in the WT *Neurospora* strain. The proteome-wide relative protein abundances (iBAQ values) were determined by MS. (**B**) Line plots showing the correlations between protein length and abundance as a function of codon usage bias measured by gene tAI values in different eukaryotic organisms. Proteome-wide protein abundance data are described and cited in Materials and Methods. All genes with detected protein levels were ranked by their tAIs, and the Pearson's correlation coefficients were calculated in continuous scanning windows from low to high tAIs. Each scanning window has 500 genes for *N. crassa*, *S. cerevisiae*, *D. melanogaster*, *C. elegans* and 1000 genes for different tissues of *M. musculus*. Methods used to quantify the relative protein levels are indicated.

To determine the potential role of codon usage in determining the levels of proteins of different sizes, we performed MS analysis of the WT *N. crassa* whole-cell extracts and determined the relative abundances of ∼3000 proteins (Supplementary Table S1). As expected, a negative correlation (Pearson's correlation coefficient R = −0.37) was observed between protein abundance and protein length proteome-wide (Figure 1A, left). However, when the analysis was only limited to genes with strong codon usage biases (codon bias index (CBI) > 0.5, genes with a strong preference for optimal codons) (63,78), the negative correlation between protein abundance and protein length was mostly abolished (*R* = −0.08) (Figure 1A, center). When genes were limited to those with low CBI values (<0.2, genes with weak codon usage biases), the negative correlation between protein abundance and protein length became stronger (*R* = −0.47) (Figure 1A, right). We also quantified gene codon usage bias using the tRNA adaptation index (tAI), a measure that takes into account tRNA concentrations and the efficiencies of codon–anticodon pairing (79). As gene tAI values increase (i.e. codon usage becomes more optimal), the negative correlation between protein abundance and protein length in a scanning window progressively weakened. The same observation was seen for the MS results obtained in this study using the iBAQ method or our previous result using the emPAI method (13) (Figure 1B).

To determine whether the codon usage effect observed above in *Neuropsora* is conserved in other eukaryotes, we determined the correlations between protein abundance and protein length as a function of gene tAI values in *S. cerevisiae*, *D. melanogaster, C. elegans* and different mouse tissues using previously reported proteomic MS data (74–77). As in *N. crassa*, there are negative correlations between protein abundances and protein lengths in all these eukaryotic organisms, and the negative correlations progressively weaken as codon usage becomes more optimal (Figure 1B), regardless of the protein quantification methods used. These results suggest that optimal codon usage can counter the negative impact of CDS length on protein expression to allow large proteins to be efficiently expressed.

To determine whether the codon usage and length-dependent effect on protein levels is due to the regulation at mRNA level, we determined the correlations between mRNA levels and CDS lengths as a function of gene codon usage using our RNA-seq results from the *Neurospora* WT strain (see below). As shown in Supplementary Figure S3, codon usage does not appear to affect the negative correlation between mRNA level and CDS, suggesting that the codon usage effect on the correlation between protein abundance and length is likely regulated at the translational level.

### The codon usage effects on mRNA translation and ribosome density are CDS length-dependent

**Figure 2.**
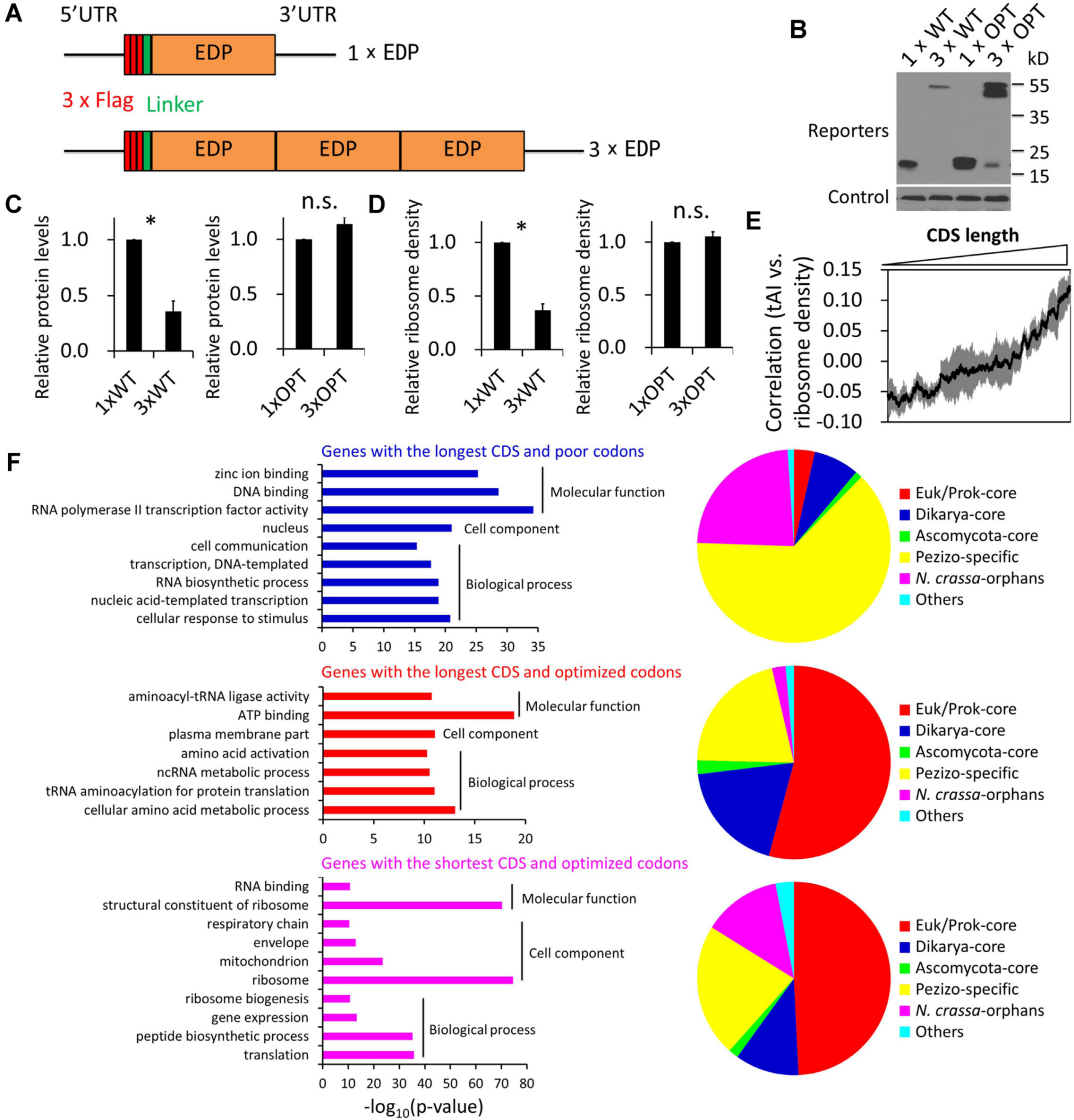
CDS length-dependent codon usage effect on protein expression and the separation of protein functions based on codon usage and CDS length. (**A**) Graphical depiction of the constructs used for the expression of the WT or OPT EDP reporters. The codon sequences for the N-terminal 3× Flag tag, the linker (8 × Gly) at the N terminus, the 5′-UTR and 3′-UTR are identical in all constructs. (**B**) Western blot analysis showing that the expression of the indicated EDP reporters. A nonspecific constitutive band detected by the anti-Flag antibody was used as the control. (**C**) Densitometric analyses of the relative EDP protein levels from three independent experiments. The WT and OPT reporters are compared separately and the level of 1× WT/OPT EDP protein was set as 1.0 for each panel. (**D**) Comparison of the relative ribosome density levels between the indicated reporter mRNAs. The relative ribosome density was measured by the relative RPFs normalized by the relative mRNA levels detected by qRT-PCR. The WT and OPT reporters are compared separately and the 1× WT/OPT EDP ribosome density level was set as 1.0 for each panel. Data in panel C and D are means with standard deviations (SD) (*n* = 3). * indicates *p* < 0.05 as determined by Student's two-tailed *t* test. n.s., not significant. (**E**) A line plot showing the correlations between codon usage (gene tAI) and ribosome density in 500-gene scanning windows as a function of CDS length (from short to long). (**F**) Left panels: Gene functional enrichment analyses showing the separation of protein functions based on codon usage and CDS length. Genes in the *N. crassa* genome were divided into different groups based on codon usage and CDS length (see Supplementary Figure S5). The most statistically enriched GO categories for genes in the indicated groups were shown. See Supplementary Table S2 for the complete list. Right panels: Pie charts showing the proportions of genes (corresponding to the left panels) classified into mutually exclusive lineage specificity groups based their conservation in other organisms.

To confirm the codon usage effect on protein abundance in a CDS length-dependent manner, we created four N-terminally Flag-tagged reporter constructs with different codon usage biases and CDS lengths for expression (Figure 2A). The CDS regions of the reporters correspond to the sequence of *NCU05784*, which encodes a small (125 aa), hypothetical protein, which we named elongation-dependent phosphorylated protein (EDP, see below). To determine the effect of CDS length on protein expression independent of codon usage, we created the 1 × EDP and 3× EDP (3 tandem EDP repeats) constructs (Figure 2A). The EDP open reading frames are composed of either the WT codons or OPT codons. These expression constructs were targeted to the *csr-1* locus in the *N. crassa* genome. Homokaryotic transformants containing each reporter construct were obtained. For the WT codon usage constructs, 1× EDP was produced in a considerably higher level than 3× EDP (Figure 2B and C). Codon optimization resulted in higher protein levels for both 1× EDP and 3× EDP, but the codon optimization effect on protein up-regulation was much more robust for 3× EDP than for 1× EDP such that their abundances were comparable after codon optimization (Figure 2B, C and Supplementary Figure S4A). Thus, consistent with the bioinformatics analysis results, codon usage has differential effects on protein expression in a CDS length-dependent manner: Optimal codon usage preferentially allows large proteins to be efficiently expressed, and non-optimal codon usage has a more potent inhibitory effect on the expression of larger proteins than smaller ones.

To determine whether codon usage affects translation efficiency in a CDS length-dependent manner, we performed ribosome profiling using the WT strains expressing the different reporter proteins. Ribosome profiling is a powerful approach for studying mRNA translation dynamics *in vivo* as it provides codon-level resolution of ribosome locations and ribosome occupancy on mRNAs (21,80,81). Ribosome density on a given mRNA can be determined by the number of its RPFs normalized by its RNA level within the CDS region, and can reflect the ribosome flux of that mRNA (80). The relative ribosome density on the WT 3× EDP mRNA was significantly lower than that on the WT 1× EDP mRNA (Figure 2D and Supplementary Figure S4B). Note that the CDS regions of these two mRNAs have the identical codon usage profile. In contrast, the relative ribosome densities were comparable for the optimized (1 × OPT and 3 × OPT) mRNAs (Figure 2D and Supplementary Figure S4B). These results suggest that non-optimal codon usage preferentially inhibits translation of mRNAs with longer CDS regions. In addition, despite of the higher EDP protein levels for the OPT reporters, their ribosome densities were actually lower than that for the 1× WT reporter (Supplementary Figure S4B). Because we previously showed that optimal codons can dramatically reduce ribosome densities on mRNAs in *Neurospora* due to increased elongation speed (21,23), this result suggests that the reduction of ribosome density caused by increased elongation speed due to codon optimization more than counters the increase of ribosome density caused by increased translation initiation efficiency.

To examine the CDS length-dependent effect on translation genome-wide, we determined the ribosome densities within CDS regions of all predicted *Neurospora* genes using the ribosome profiling results of the WT strain and calculated the correlations between tAIs and ribosome densities as a function of CDS lengths. It is important to note that codon usage has a major impact on elongation rates and optimal codons can dramatically reduce ribosome densities on mRNAs in *Neurospora* (21,23). Thus, ribosome density measurement will overestimate the ribosome flux for mRNAs with poor codon usage and underestimate the flux for those with optimal codon usage. The correlations between gene tAIs and ribosome densities are weakly negative for short mRNAs (Figure 2E). As CDS length increases, however, the correlations gradually become positive, suggesting that optimal codon usage of long mRNAs positively correlates with ribosome density (Figure 2E). Because optimal codons result in fast elongation rates which lower ribosome density, the weak positive correlation actually indicates a strong positive effect of optimal codon usage on ribosome flux. Thus, codon usage affects ribosome flux on mRNAs in a CDS length-dependent manner: codon optimality preferentially enhances translation efficiency/ribosome flux of long mRNAs. These results also indicate the existence of a feedback mechanism from translation elongation to regulate translation initiation.

### Codon usage and CDS length-dependent separation of gene functions in the genome

Because codon usage differentially affects protein expression level in a CDS length-dependent manner, we hypothesize that large proteins with critical functions would have optimal codon usage profiles to allow their efficient synthesis. To examine this, we grouped all predicted *N. crassa* genes based on their CBI values and CDS lengths and performed gene functional enrichment analyses for four different groups of genes (each with 1000 genes) (Supplementary Figure S5): (i) those with the longest CDS regions among those with strong codon usage biases (CBI ≥ 0.3), (ii) those with the longest CDS regions among those with non-optimal codon usage biases (CBI ≤ 0.15), (iii) those with the shortest CDS regions among those with strong codon usage biases and (iv) those with the shortest CDS regions among those with non-optimal codon usage biases. There was no significant functional enrichment (*P*-value < 1e−10) for genes in group (iv). In contrast, many genes with similar functions or in the same biological process were significantly enriched in the other three groups (Figure 2F, left panels and Supplementary Table S2). As predicted, the genes with long CDS regions and optimal codon usage are enriched for functional categories associated with essential cellular processes such as amino acid activation and amino acid metabolic process, tRNA aminoacylation, plasma membrane components, and non-coding RNA metabolic process (Figure 2F left panels and Supplementary Table S2). In contrast, the genes with the long CDS regions and poor codon usage profiles are mostly enriched for functional categories involved in the responses to environmental stimulus, cell communication, and transcriptional regulation (Figure 2F left panels and Supplementary Table S2). The genes with the shortest CDS regions and optimal codons are significantly enriched for functional categories related to translation, ribosomal proteins and ribosome biogenesis, mitochondrial components and respiratory chain (Figure 2F, left panels and Supplementary Table S2).

We also classified the genes of the three groups in Figure 2F, left panels into six mutually exclusive lineage specificity groups based their conservation in other organisms (82): (i) eukaryote/prokaryote-core (genes with homologs in non-fungal eukaryotes and/or prokaryotes), (ii) dikarya-core (genes with homologs in *Basidiomycota* and *Ascomycota* species), (iii) *Ascomycota*-core, (iv) *Pezizomycotina*-specific, (v) *N. crassa*-orphan genes and (vi) others (genes with homologs identified in prokaryotes or non-fungal eukaryotes in addition to *Pezizomycotina*, but not in members of the *Basidiomycota*, *Saccharomycotina* or *Taphrinomycotina*). As shown in the right panels of Figure 2F, the genes with long CDS regions and optimal codon usage are mostly genes in class (i) and (ii), indicating that they are conserved beyond fungi and likely have functions critical for cell survival. In contrast, the genes with long CDS regions and poor codon usage profiles are mostly *N. crassa*-specific and *Pezizomycotina*-specific. Systematic deletion studies of *Neurospora* genes previously revealed that the essential genes are mostly genes that are conserved beyond fungi, while the *Neurospora*-specific genes are not critical for cell survival (65). Together, these results are consistent with our hypothesis that optimal codon usage is a mechanism that allows large proteins required for critical cellular functions to be efficiently produced.

### Ribosome stalling at a stage between pre-accommodation and pre-translocation induces eIF2α phosphorylation

**Figure 3.**
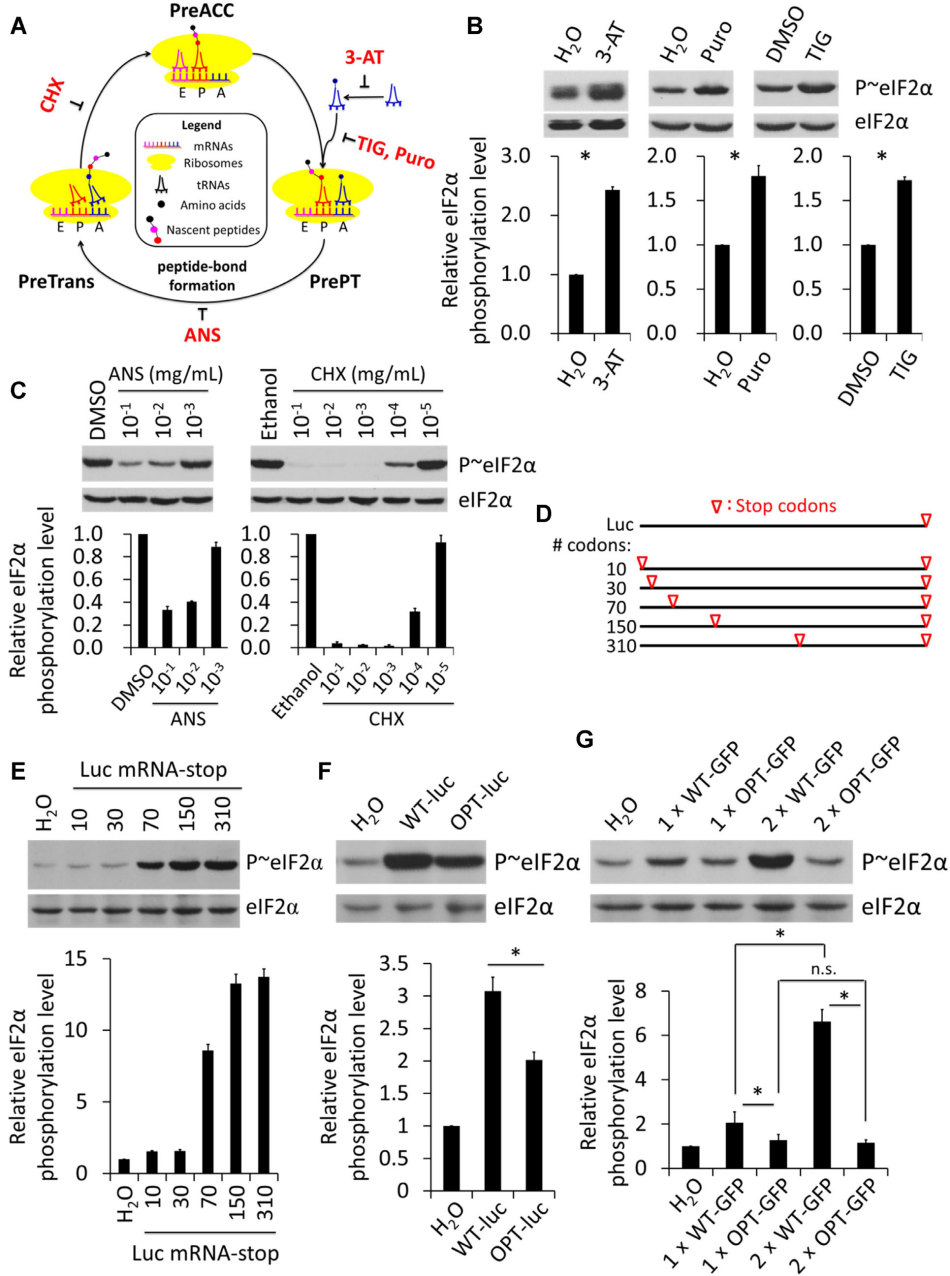
eIF2α phosphorylation can be induced by either translation inhibitors or poor codon usage in a CDS length-dependent manner. (**A**) Schematic representation of the eukaryotic translation elongation cycle and the effects of the indicated chemical inhibitors of translation. PreAcc, pre-accommodation; PrePT, pre-peptide bond formation; PreTrans, pre-translocation. (**B**, **C**) Western blot analyses and their quantifications showing the relative levels of eIF2α phosphorylation and eIF2α after treating the WT *Neurospora* cultures with the indicated inhibitors for 20 min. For experiment in each panel, equal amount of solvent used for dissolving the corresponding chemical was used as control. (**D**) Schematic diagram of the luciferase mRNA-stop constructs used for *in vitro* transcription to synthesize luciferase mRNAs with an in-frame stop codon at different indicated codon positions after the start codon within its CDS. (**E−G**) Western blot analyses and their quantifications showing the relative levels of eIF2α phosphorylation and eIF2α in the *Neuropsora in vitro* translation products. The indicated mRNA templates were translated in the *Neurospora in vitro* translation extracts for 15 min before the reactions were stopped. Data are means with SD (*n* = 3). *P < 0.05, as determined by Student's two-tailed *t* test.

The effect of codon usage on ribosome flux suggests that codon usage-dependent elongation can feedback to regulate translation initiation under nutrient replete growth conditions. Phosphorylation of eIF2α is an important regulatory mechanism of translation initiation, and is known to be induced by many types of stress conditions to result in global inhibition of translation initiation of many mRNAs (42–44,50). Since codon usage has been shown to play an important role in determining elongation speed and rare codons cause ribosome pausing with empty A site in *Neurospora* (21,23), we first examined whether ribosome stalling can trigger eIF2α phosphorylation by using different pharmacological inhibitors that can block translation elongation at different steps of the eukaryotic translation elongation cycle (Figure 3A).

We first treated *Neurospora* cultures with 3-AT, a competitive inhibitor of the product of *his-3* gene, which results in accumulation of uncharged tRNAs and cellular amino acid starvation. This treatment resulted in a significant elevation of eIF2α phosphorylation (Figure 3B). Puromycin (Puro, tyrosyl-tRNA-like) and tigecycline (TIG, tetracycline-like) can cause ribosome stalling at the stage between pre-accommodation and pre-translocation in the translation elongation cycle (83–85) (Figure 3A). Treatment of *Neurospora* cultures with either agent also enhanced eIF2α phosphorylation *in vivo* (Figure 3B). In contrast, treatments with anisomycin (ANS) and cycloheximide (CHX), which inhibit peptide bond formation and translocation, respectively, resulted in dramatic dose-dependent decreases of eIF2α phosphorylation (Figure 3C). These results suggest that ribosome stalling at the stage between pre-accommodation and pre-translocation but not at other stages of the elongation cycle induces eIF2α phosphorylation. This phenomenon may be caused by distinct ribosome conformations when ribosomes stall at different functional states. Consistent with this notion, alteration of ribosome conformation at distinct elongation states caused by CHX, TIG and ANS treatments was previously demonstrated experimentally (84). During the preparation of this manuscript, a similar conclusion on the effects of some of these inhibitors was also reached in yeast (86).

### Induction of eIF2α phosphorylation by mRNA translation is dependent on CDS length and codon usage

Although eIF2α phosphorylation can be induced by treatment of cultures with translation inhibitors, it is not clear whether it can be regulated by codon usage or CDS length under normal growth (nutrient replete) conditions. To examine these possibilities, we took advantage of the *Neurospora* cell free *in vitro* translation system that was previously shown to accurately reflect protein translation *in vivo* (21,87,88). Cellular mRNAs were depleted from this system by micrococcal nuclease digestion so that the translation of a single species of mRNAs and its impact on eIF2α phosphorylation can be examined. We synthesized a series of capped and polyadenylated WT *luciferase* (*luc*) mRNAs with an in-frame stop codon at different positions ranging from the 10th codon to 310th codon from the start codon (Figure 3D). Quantification of eIF2α phosphorylation after translation of these mRNAs revealed that there was a CDS length-dependent effect on eIF2α phosphorylation: mRNAs with long CDS regions result in higher level of eIF2α phosphorylation than those with short CDS regions (Figure 3E).

To determine whether eIF2α phosphorylation is dependent on codon usage, we evaluated the eIF2α phosphorylation level in the presence of the WT *luc* mRNA or the OPT *luc* mRNA. As expected, the translation of the WT mRNA resulted in a significantly higher level of eIF2α phosphorylation than the translation of the OPT version (Figure 3F and Supplementary Figure S6A). To confirm that this result is not gene specific, we synthesized and translated the WT or OPT versions of mRNAs encoding one or two copies of the *GFP* CDS regions (1 × GFP and 2 × GFP, respectively) (Supplementary Figure S6B). Both of the WT *GFP* mRNAs induced significantly higher levels of eIF2α phosphorylation than the OPT mRNAs (Figure 3G). Strikingly, the induction of eIF2α phosphorylation by the WT 2× GFP was much higher than that by the WT 1× GFP, while the OPT 2× GFP mRNAs had little effect on eIF2α phosphorylation compared to the OPT 1× GFP. Together, these results demonstrate that translation elongation can induce eIF2α phosphorylation in a CDS length-dependent and codon usage-dependent manner in the absence of translation stress. Thus, codon usage and CDS length can potentially regulate translation initiation by affecting eIF2α phosphorylation.

### Loss of eIF2α kinase CPC-3 preferentially up-regulates protein expression for mRNAs with long CDS and poor codon usage

**Figure 4.**
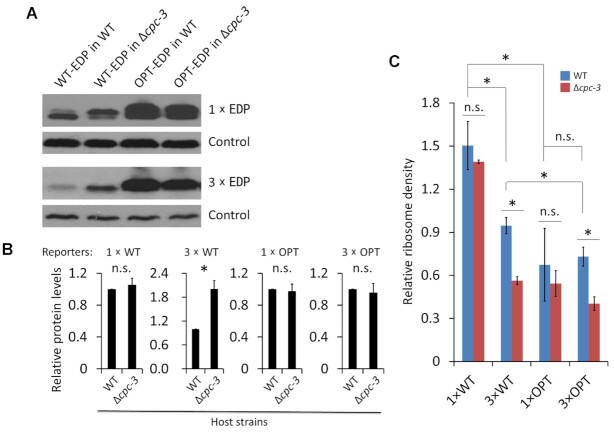
Codon usage and CDS length-dependent effect on protein expression level in the Δ*cpc-3* strain. (**A**) Western blot analysis results showing the expression levels of the Flag-tagged 1× EDP (WT or OPT) and 3× EDP (WT or OPT) reporters in the WT or Δ*cpc-3* strains. A nonspecific constitutive band detected by the anti-Flag antibody was used as the control. (**B**) Densitometric analyses of the western blot results of the EDP levels from three independent experiments described above. The EDP protein level in the WT strain for each panel was set as 1.0. (**C**) Comparison of the relative ribosome density levels between the indicated reporter mRNAs in the WT strain and the Δ*cpc-3* strain. The relative ribosome density was measured by the relative RPFs normalized by the relative mRNA levels detected by qRT-PCR. Data in panel B-C are means with SD (*n* = 3). * indicates *P* < 0.05 as determined by Student's two-tailed *t* test. n.s., not significant.

eIF2α phosphorylation does not have to cause global down-regulation of translation initiation (44,50). Our findings that eIF2α phosphorylation depends on codon usage and CDS length in the absence of translation stress suggest that this is an elongation-dependent feedback mechanism that may alter translation efficiency of specific mRNAs. We hypothesize that long mRNAs and those enriched with rare codons cause local accumulation of phosphorylated eIF2α, resulting in specific rather than a general suppression of mRNA translation. *cpc-3* (*cross pathway control-3*, NCU01187) encodes the *Neurospora* homolog of the yeast and mammalian GCN2, and is the only known kinase responsible for eIF2α phosphorylation in *Neurospora* (52). As expected, eIF2α phosphorylation is completely abolished in the Δ*cpc-3* strain (Supplementary Figure S1). We compared the expression of the four EDP reporters described above in the WT and Δ*cpc-3* strains. Although the deletion of *cpc-3* did not affect the expression levels of the WT or OPT 1×EDP or OPT 3×EDP, it significantly increased the protein level of the WT 3×EDP (Figure 4A, B and Supplementary Figure S7). These results suggest that eIF2α phosphorylation preferentially inhibits the translation of long CDS mRNAs with poor codon usage, resulting in their specific translation inhibition rather than a general translation inhibition. Although deletion of *cpc-3* increased the protein level of WT 3× EDP, its level was still lower than that of the WT 1xEDP level (Supplementary Figure S7), suggesting that the effect of CDS length on protein abundance is determined by both CPC-3-dependent and CPC-3-independent mechanisms. A CPC-3-independent mechanism may be involved in the negative influence of CDS length on translation due to less efficient ribosome recycling for mRNAs with long CDS than for short CDS mRNAs (39,41). It should also be noted that the negative effect of ‘CDS length’ on protein level depends on codon usage, because unlike the WT reporters, the protein levels of the 1× OPT and 3× OPT EDP are comparable (Figure 4A and Supplementary Figure S7), which is consistent with the proteomic data analysis results (Figure 1). This result indicates that the negative effect of CDS length on protein production is attenuated for mRNAs with optimal codon usage.

### Deletion of *cpc-3* resulted in a decrease of ribosome density of the reporter mRNAs with long CDS

To understand how CPC-3 influences translation *in vivo*, we also performed ribosome profiling experiment in the Δ*cpc-3* strains expressing the different EDP reporters under nutrient replete condition and compared the relative ribosome density of the EDP reporter mRNAs in the WT and Δ*cpc-3* strains by normalizing the number of RPFs on CDS regions with mRNA levels. Consistent with the result in Figure 2D, the relative ribosome density of 3× WT EDP was significantly decreased compared to that of the 1× WT EDP in the WT strain, while that of 3 × OPT EDP is comparable to that of 1× OPT EDP (Figure 4C). In addition, the ribosome densities of 1× and 3× WT EDP mRNAs were both significantly higher than that of their OPT counterparts in the WT strain (Figure 4C), indicating that codon optimization results in faster translation elongation speed, which reduced ribosome density despite the strong up-regulation of OPT mRNA translation (21). Surprisingly, compared to the WT strain, the relative ribosome density of the 3× WT EDP but not 1× WT EDP was significantly decreased in the Δ*cpc-3* strain (Figure 4C). The up-regulation of 3× WT EDP protein level but a decrease of ribosome density on its mRNA in the Δ*cpc-3* strain suggests that, in addition to its role on translation initiation, CPC-3 may also have a role that preferentially inhibits translation elongation on mRNAs with long CDS and poor codon usage. As a result, the increase of elongation speed on 3× WT EDP mRNA in the Δ*cpc-3* strain more than counters the effect of the increase of translation initiation, resulting in a decrease of ribosome density.

### CPC-3 slows down translation elongation rate in a codon usage-dependent manner

**Figure 5.**
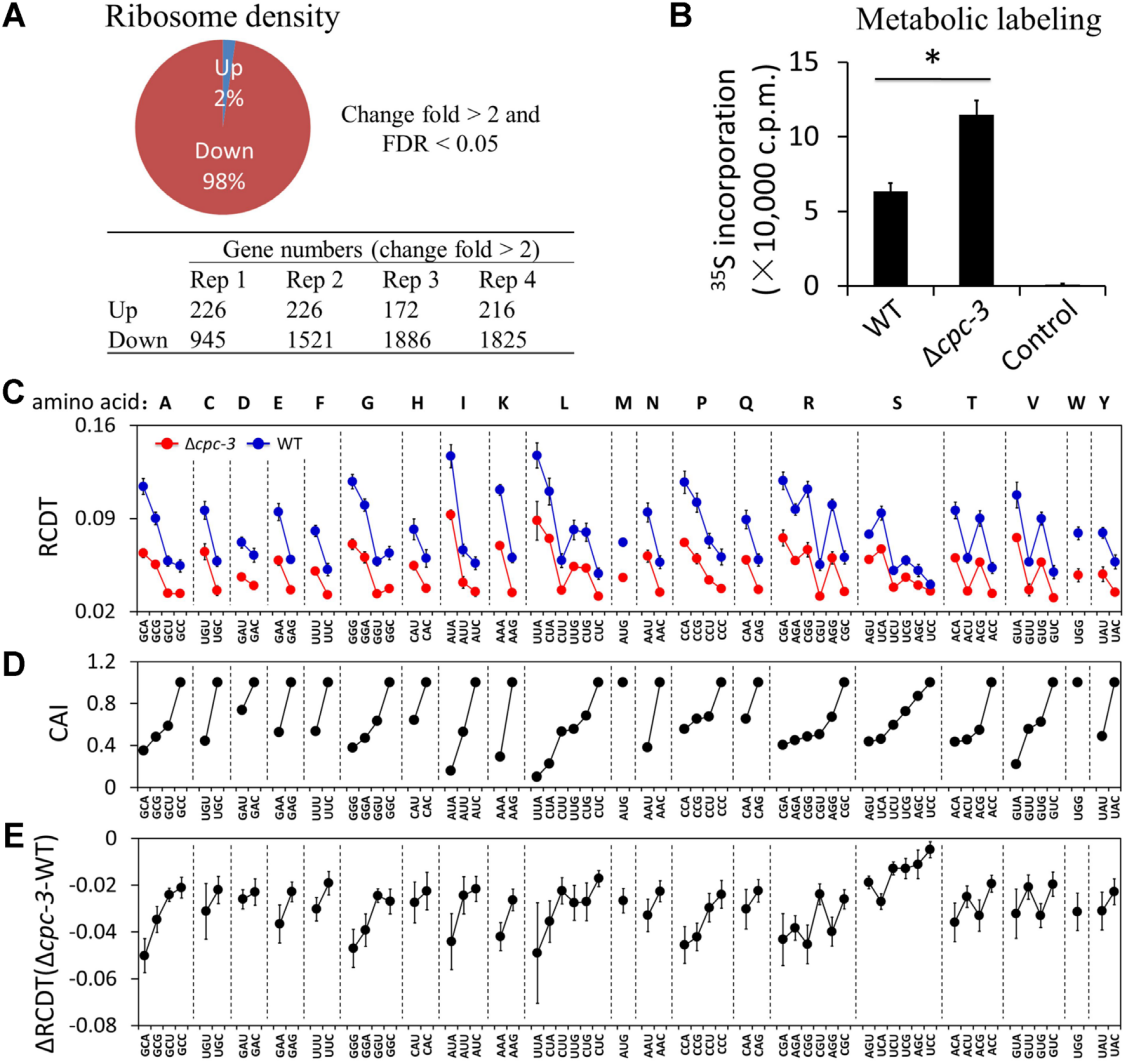
Ribosome profiling results show that CPC-3 slows down codon decoding rates in a codon usage-dependent manner. (**A**) Upper panel: A pie chart showing the percentages of mRNAs with reduced (red) or increased (blue) ribosome densities on mRNAs in the Δ*cpc-3* strain comparing to the WT strain. Genes with significantly increased and decreased ribosome density were identified by using the following cut-off: the change fold of the average of the four replicates >2 and FDR <0.05. The *P*-values for the four replicates were determined by Student's two-tailed *t* test, which were further adjusted by FDR. Lower panel: A table showing the numbers of genes with change fold of ribosome density >2 (up- or down-regulated) in the four replicates. (**B**) Metabolic labeling experiment indicated an increased global protein synthesis rate in the Δ*cpc-3* strain. ^35^S-Methionine was added into the cultures for 45 min before sample collection. For the control culture, CHX (a final concentration of 0.1 mg/ml) was added 2 min before adding ^35^S-methionine. Data are means with SD (*n* = 3). * *P* < 0.05; as determined by Student's two-tailed *t* test. (**C**) Comparison of RCDTs in the WT and Δ*cpc-3* strains (*n* = 3). (**D**) The CAI values of the corresponding codons in each codon family. (**E**) Quantification of the differences of RCDTs between the Δ*cpc-3* strain and the WT strain showed that CPC-3 preferentially impacts the elongation rates of rare codons in each codon family. Data are means with SD (*n* = 3).

To confirm our conclusion above and determine the effect of CPC-3 genome-wide, we calculated the gene-specific ribosome densities for all predicted *Neuropsora* genes using the ribosome profiling and accompanying RNA-seq results of the WT and Δ*cpc-3* strains. We found that among genes with more than 2-fold changes (FDR < 0.05) in ribosome density in the Δ*cpc-3* strain compared to the WT strain, 98% genes had a decreased ribosome density whereas only 2% had an increased ribosome density (Figure 5A). To further confirm this result, we also performed polysome profiling experiments, which showed that the ratio of polysomes/monosome in the Δ*cpc-3* strain was lower than that in the WT strain (Supplementary Figure S8A and B), consistent with the ribosome profiling result.

The global decrease of ribosome densities on mRNAs in the Δ*cpc-3* strain is unexpected due to the well-established role of CPC-3 and eIF2α phosphorylation in inhibiting translation initiation. To confirm the inhibitory role of CPC-3 and eIF2α phosphorylation in translation, we performed ^35^S-methionine pulse labeling experiment to compare the overall translation efficiencies between the WT and Δ*cpc-3* strains grown at 30°C. As expected, the Δ*cpc-3* strain had a significant increase of ^35^S-methionine incorporation level than the WT strain (Figure 5B), indicating that CPC-3 and eIF2α phosphorylation indeed inhibit general translation efficiency in *Neurospora*. Just like what we found for the 3× WT EDP reporter mRNA, the increase of general translation efficiency but reduction of ribosome densities on most of mRNAs in the Δ*cpc-3* strain suggests an overall increased translation elongation rate in the Δ*cpc-3* strain that can more than counter the effect of the increase of translation initiation on ribosome density.

To determine the role of CPC-3 in translation elongation rate, we calculated the RCDTs for all 61 amino acid-encoding codons in the WT and Δ*cpc-3* strains using the ribosome profiling results. Consistent with our previous studies (21,23,58), there was a clear codon usage bias in RCDTs for all codon families in the WT strain. The most preferred synonymous codon was always the one with the lowest RCDT in each codon family, while rare codons had the highest RCDTs (Figure 5C and D). Although the codon usage biases in RCDTs did not change in the Δ*cpc-3* strain, RCDTs were reduced for all codons (Figure 5C and D). This indicates that there was a global increase in translation elongation rate in the Δ*cpc-3* strain, resulting in the decreased ribosome density on most mRNAs despite the increase of translation initiation. We next examined whether the effect of CPC-3 on translation elongation is codon usage-dependent. We observed that the decrease of RCDTs in the Δ*cpc-3* strain compared to the WT strain was always greater for the rare codons than for the most preferred codons in all codon families (Figure 5E), indicating that CDC-3 preferentially slows translation elongation at rare codons.

**Figure 6.**
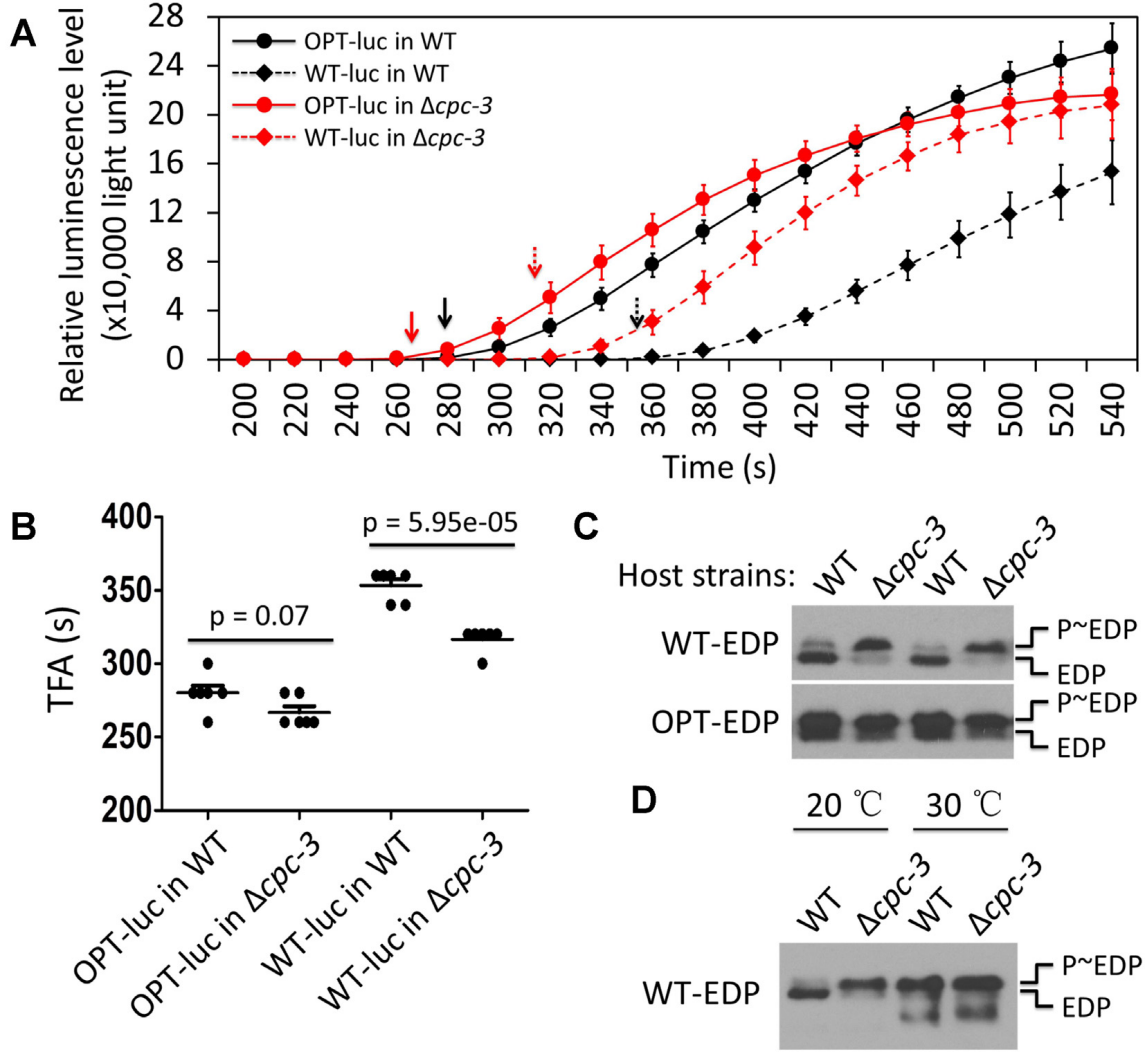
Codon usage-dependent increase of translation elongation rate in the Δ*cpc-3* strain demonstrated by *in vitro* translation assay and an *in vivo* protein phosphorylation reporter. (**A**) *In vitro* translation experiments of the indicated strains using the *Neurospora* cell-free translation system showed the comparison of the temporal profiles of luciferase signal level of different luciferase mRNAs at 26°C. The arrows indicate the TFAs of the luciferase signal for indicated mRNAs. Data are represented as mean ± SD. (**B**) Scatter plots comparing the TFAs between the indicated *Luc* mRNAs in the WT or Δ*cpc-3* lysates (n = 6). Means indicated by horizontal bars. P-values was shown, as determined by Student's two-tailed *t* test. (**C**) Western blot analysis results showing that the EDP phosphorylation profiles of the indicated reporters were determined by codon usage and host strains. The phosphorylated and un-phosphorylated EDP bands were indicated. (**D**) Western blot analysis results showing that high temperature promotes the EDP phosphorylation of the WT-EDP reporter in the WT strain.

To further confirm this conclusion, we utilized the *Neurospora* cell-free translation system and the WT and OPT luciferase reporters, which was previously used to demonstrate the codon usage effect on elongation speed (21). Because luciferase is known to be folded co-translationally and becomes functional within a few seconds after the completion of translation, the time of first appearance (TFA) values of luciferase signal for the WT and OPT *luc* mRNAs reflect differences in translation elongation rates (21,58,89). In addition, translation initiation time was previously estimated to be less than several seconds (37,47,90). Thus, the TFA changes should reflect changes in elongation rates. Similar to what we reported previously (21), the TFA of the OPT *luc* mRNA was significantly shorter than that of the WT *luc* in the WT extracts (Figure 6A and B). In the Δ*cpc-3* extracts, the TFA values of both WT and OPT *luc* mRNAs were reduced, confirming the effect of CPC-3 on translation elongation. Importantly, the impact of loss of CPC-3 on translation elongation rate was clearly codon usage-dependent: The TFA was significantly faster (by ∼40 s) for the WT *luc* mRNA in the Δ*cpc-3* extracts than in the WT extracts, but for the OPT *luc* mRNA, the TFA was only marginally reduced in the Δ*cpc-3* extracts and was not statistically different from that in the WT extracts (Figure 6A and B).

When the WT 1× EDP reporter was expressed in the WT strain, we noticed that there were several protein species with different mobilities in SDS-PAGE gel (Figure 4A). Phosphatase treatment of the protein extracts indicated that the bands that migrated more slowly were phosphorylated EDP (Supplementary Figure S9). EDP was mostly in the hypo-phosphorylated form in the WT strain but was mostly hyper-phosphorylated in the Δ*cpc-3* strain (Figure 4A and 6C). The expression of the OPT 1 × EDP, however, resulted in hyper-phosphorylation of EDP in both of the WT and Δ*cpc-3* strains (Figure 6C). This codon usage-dependent protein phosphorylation profile change is very similar to what we previously observed when the codon usage profiles of genes encoding circadian clock proteins FRQ and PER from *Neurospora* and *Drosophila*, respectively, were changed (16,30,63). These results suggest that the EDP phosphorylation profile is affected by the co-translational protein folding process that is sensitive to translation elongation rate regulated by codon usage. Rapid translation elongation caused by either codon optimization or deletion of *cpc-3* results in altered EDP structure that promotes its phosphorylation. To further confirm this, we compared the WT 1 × EDP expression profiles for cultures grown at 20 and 30°C. We have shown previously that higher temperature increases translation elongation rate (21). As expected, in the WT strain, the protein expressed from WT 1× EDP was hypo-phosphorylated at 20°C but became hyper-phosphorylated at 30°C (Figure 6D). On the other hand, EDP expressed from WT 1 × EDP mRNA was hyper-phosphorylated at both temperatures in the *cpc-3* strain. Together, these results show that CPC-3 not only regulates translation initiation via eIF2α phosphorylation but also translation elongation in a codon usage-dependent manner. Thus, CPC-3 plays an important role in determining the codon usage effect on elongation speed so that optimal codons are decoded much faster than rare codons. Our results here also caution the use of ribosome density as a reflection of translation efficiency because elongation rate can have a major impact on ribosome density.

### CPC-3 influences translation kinetics in a CDS length-dependent manner

**Figure 7.**
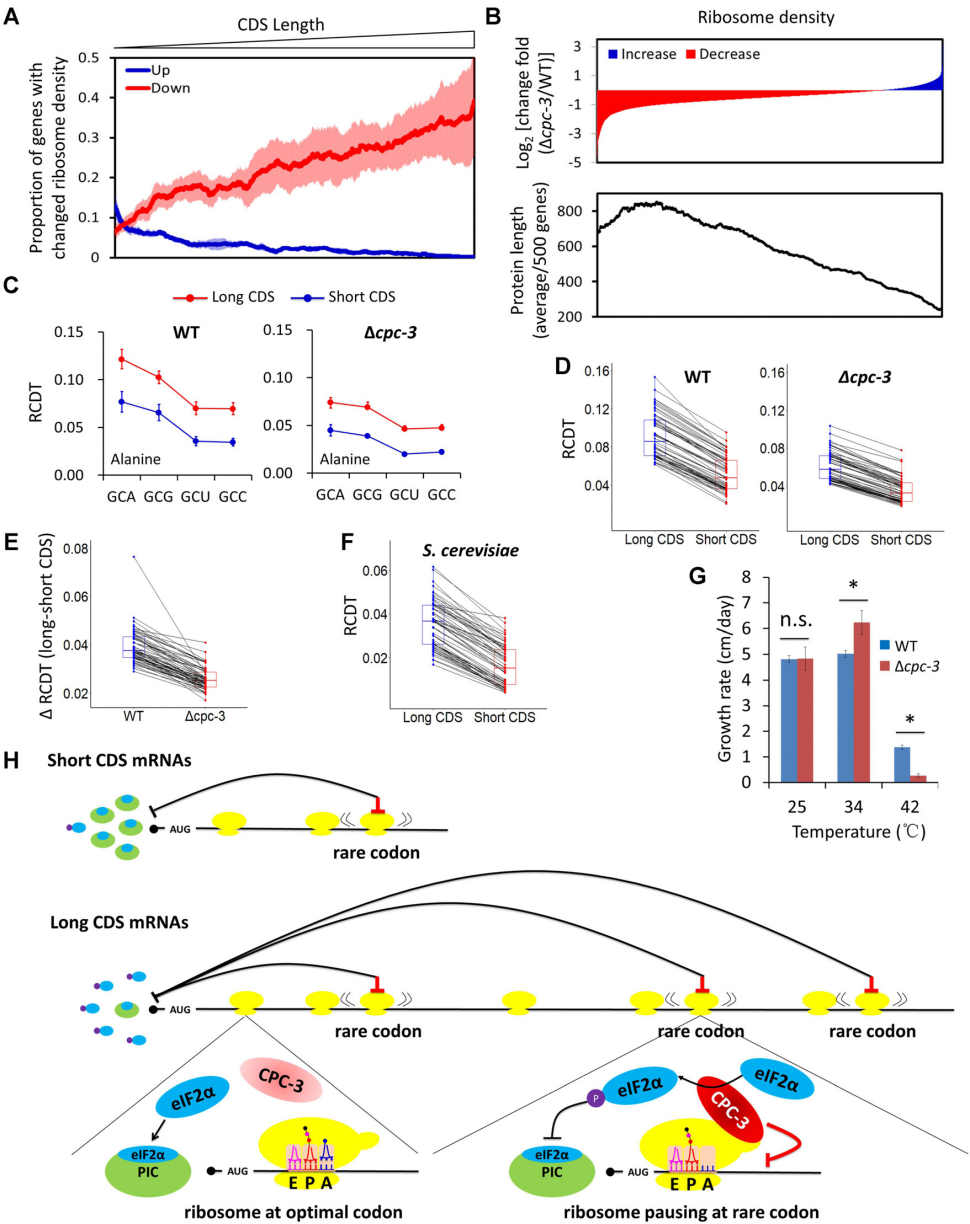
CPC-3 regulates CDS length-dependent codon decoding rates. (**A**) Line plots showing that the proportions of genes with decreased ribosome density in the Δ*cpc-3* strain (change fold of Δ*cpc-3*/WT < 0.5) increased as protein length increased. Genes were ranked by their CDS lengths from short to long and the proportions of genes with decreased/increased ribosome density in a scanning 500 gene-window were calculated and plotted. Data are means with SD (*n* = 4). Shaded areas indicate error bars in each scanning window. (**B**) Genes with decreased ribosome density preferentially code large proteins while genes with increased ribosome density prefer to code shorter proteins. Top panel: genes were ranked by ribosome density changes [log_2_(change fold of Δ*cpc-3*/WT)] from the greatest decrease to the greatest increase. Bottom panel: the averages of protein lengths in 500-gene scanning windows corresponding to the ribosome density change above were calculated and plotted. (**C**) The RCDTs of the alanine codons within mRNAs with CDS >600 aa and those <300 aa in the WT and Δ*cpc-3* strains. Results for all other codons are shown in Supplementary Figure S11. (**D**) Pairwise comparison of RCDTs of all codons in the long and short CDS mRNAs in the WT and Δ*cpc-3* strains. The same codons were linked by black lines. (**E**) The differences of RCDTs of all codons (pairwise comparison) between long and short CDS mRNAs [ΔRCDT (long-short CDS)] are always larger in the WT strain than that in the Δ*cpc-3* strain. (**F**) Pairwise comparison of RCDTs of all codons in long and short CDS mRNAs in *S. cerevisiae*. The previously published ribosome profiling and RNA-seq data were used for this analysis (91). (**G**) Comparison of daily growth rates of the WT and Δ*cpc-3* strains by race tube assay at 25, 34 and 42°C. The asterisk indicates *P* < 0.05 (*n* = 6) as determined by Student's two-tailed *t*-test. (**H**) A schematic illustration of a model explaining the mechanism of translation elongation feeding back to regulate translation initiation and elongation speed in a codon usage and CDS length-dependent manner. Bottom left: ribosome does not pause at optimal codons during elongation. Bottom right: Rare codons cause ribosome pausing during elongation, which potentially promotes the interaction between CPC-3 and ribosomes, resulting in CPC-3 activation and phosphorylation of eIF2α-GDP. The phosphorylated eIF2α-GDP prevents the recycling of eIF2α-GDP to become eIF2α-GTP, thus inhibiting the formation of pre-initiation complex (PIC) and therefore translation initiation. Translation of an mRNA with a short CDS triggers less rare codon-mediated ribosome pausing, thus less CPC-3 activation and less the eIF2α phosphorylation-mediated inhibition of initiation. Translation of an mRNA with a long CDS potentially triggers more rare-codon mediated ribosome pausing events, resulting in high local concentration of phosphorylated eIF2α-GDP, which inhibits ribosome recycling and translation re-initiation. In addition, CPC-3 also inhibits translation elongation rate in a codon usage-dependent manner so that codon decoding rates for optimal codons are faster than those for non-optimal codons.

Because the CPC-3-mediated eIF2α phosphorylation is dependent on both codon usage and CDS length, we examined whether the role of CPC-3 on translation kinetics is also influenced by CDS length. We calculated the proportions of genes with up-regulated (change fold of Δ*cpc-3*/WT > 2) or down-regulated (change fold of Δ*cpc-3*/WT < 0.5) ribosome density in a 500-gene scanning window. After ranking genes by their CDS lengths from short to long, we found that as CDS length gradually increased, the proportions of genes with down-regulated ribosome density increased markedly (Figure 7A). In contrast, the proportions of genes with up-regulated ribosome density decreased as CDS length increased in the same corresponding window (Figure 7A). When we ranked genes by their log_2_ [change fold (Δ*cpc-3*/WT)] of ribosome density from low to high and determined their averages of CDS lengths in 500-gene scanning windows, it was clear that the genes with down-regulated ribosome density are mostly those with long CDS mRNAs (>700 aa), whereas the genes with up-regulated or unchanged ribosome density tend to be short CDS mRNAs (Figure 7B). These results are also consistent with the results of the four EDP reporters (Figure 4C), which showed that the relative ribosome density was significantly decreased for the 3× EDP mRNAs but not for the 1× EDP mRNAs in the Δ*cpc-3* strain. Together, these results suggest that the inhibitory effect of CPC-3 on translation kinetics is dependent on both codon usage and CDS length.

We then performed proteomic quantitative MS analyses to identify differentially expressed proteins between the Δ*cpc-3* strain and its complementation strain by TMT mass tagging technology (Supplementary Table S3). The result showed that, after ranking proteins by their lengths from short to long, the proportions of up-regulated proteins (FDR < 0.05) increase in a 1000-gene scanning window as protein size increases (Supplementary Figure S10A). In addition, comparison of the protein length profiles showed that the up-regulated proteins are preferentially larger proteins (with an average of 645 aa) than the predicted proteome (with an average of 450 aa) (Supplementary Figure S10B). These results suggest that CPC-3 preferentially inhibits the expression of large proteins. It should be noted, however, our MS analysis preferentially identifies abundant proteins and failed to detect the vast majority of proteins encoded by mRNAs with poor codon usage (13).

### Codon decoding rates are CDS length-dependent and are regulated by CPC-3

It was previously assumed that the same codons can be recognized and translated with similar efficiency on different mRNAs (21,22). The CDS length-dependent effect on ribosome density prompts us to examine whether codon decoding rate is also affected by CDS length. Thus, we compared RCDTs of all codons using the ribosome profiling data of the WT strain for mRNAs with long CDS regions (>600 aa) or short CDS regions (<300 aa). Remarkably, all codons have higher RCDTs for long CDS mRNAs than those of short CDS mRNAs (Figure 7C, D and Supplementary Figure S11). However, the difference of RCDT of each codon between long and short CDS mRNAs was much smaller in the Δ*cpc-3* strain than in the WT strain (Figure 7E). These results suggest that CPC-3 also regulates the CDS length-dependent effect on codon decoding rate. By analyzing the previously published ribosome profiling results in *S. cerevisia*e (91), the similar CDS length-dependent effect on codon decoding rates was also observed for all codons (Figure 7F), suggesting that the effect of CDS length on elongation rate is conserved in eukaryotes. Because of the role of elongation rate in regulating co-translational protein folding and because folding of large proteins is more complicated than folding of small proteins, the CDS length-dependent regulation on translation is likely an adaptive mechanism that slows translation elongation to facilitate optimal co-translational folding of large proteins.

### CPC-3 deletion resulted in increased sensitivity to heat shock treatment

Because translation kinetics influences co-translational protein folding (6,32,92,93), the deletion of *cpc-3* in *Neurospora* should broadly affect co-translational protein folding as indicated by the EDP phosphorylation profile change. If so, proteins in the Δ*cpc-3* strain may be more sensitive to conditions that trigger protein misfolding, resulting in impaired cell growth. To examine this possibility, we compared the growth rates of the WT and Δ*cpc-3* strains at normal growth temperatures (20–34°C) and at 42°C. The 42°C treatment induces a heat shock response in *Neurospora* and impairs cell growth. The heat shock treatment should also make nascent proteins prone to be misfolded if their co-translational folding processes are not optimal. Although the two strains had a similar growth rate at 25°C, the Δ*cpc-3* strain grew more rapidly than the WT strain at 34°C, which may be due to elevated protein translation (Figure 7G). At 42°C, however, the growth of the Δ*cpc-3* strain was almost completely inhibited whereas the WT strain still exhibited modest growth. This result indicates that the deletion of *cpc-3* causes increased sensitivity to heat shock treatment, which is consistent with the role of CPC-3 in regulating translation kinetics, which in turn influences protein folding and function.

## DISCUSSION

In this study, we showed that codon optimality regulates protein translation in a CDS length-dependent manner by regulating both translation initiation rate and elongation speed. Analyses of the proteomic results from *Neurospora*, yeast, fly,worm and mouse showed that protein abundance negatively correlates with protein length genome-wide. The negative correlation, however, is dependent on codon usage: As codon optimality increases, the negative correlation progressively weakens. The conserved nature of this observation suggests a common mechanism mediated by codon usage that regulates protein synthesis in a protein size-dependent manner. Using gene reporters with different codon usage biases and different CDS lengths, we showed that non-optimal codon usage preferentially reduced the production levels of large proteins and that optimal codon usage eliminated the length-dependent effect on protein production in *Neurospora*. Gene functional enrichment analysis showed that there is a functional separation of gene functions based on codon usage and CDS length: The genes encoding long mRNAs with optimal codons are significantly enriched for functional categories of essential cellular processes, whereas those encoding long mRNAs with non-optimal codon usage are enriched for functional categories involved in regulatory processes. These results suggest that optimal codon usage is a mechanism that permits efficient production of large proteins critical for cell survival.

Further, we showed that codon optimality regulates ribosome density and ribosome flux on mRNA genome-wide in a CDS length-dependent manner. We showed that codon usage- and CDS length-dependent eIF2α phosphorylation occurs in the absence of translation stress, suggesting a mechanism for how codon usage and CDS length regulate translation by feeding back on translation initiation (Figure 7H). We propose that rare codons cause ribosomes with empty A sites to pause. Such ribosome pausing results in CPC-3 activation and eIF2α phosphorylation. For translation of mRNAs with the same codon usage, the pausing occurs more often on long CDS mRNAs than that on short CDS mRNAs due to the existence of more rare codons, resulting in higher level of eIF2α phosphorylation, which can inhibit translation initiation by blocking the formation of the pre-initiation complex. However, as in yeast and mammals, CPC-3 may also have substrates other than eIF2α, such as the methionyl-tRNA synthetase. The phosphorylation of methionyl-tRNA synthetase has been shown to inhibit methionyl-tRNA synthetase activity, thus reinforcing the transient inhibition of translation initiation exerted by eIF2α phosphorylation (94,95). Thus, the eIF2α phosphorylation-independent function of CPC-3 may also contribute to its functions in translation.

Unlike the eIF2α phosphorylation induction under stress conditions that results in the integrated stress response and global repression of translation (42,53–56), the codon usage-dependent and CDS length-dependent induction of eIF2α phosphorylation is mRNA specific and does not cause a global increase of eIF2α phosphorylation under nutrient replete growth conditions. Indeed, we showed that the deletion of *cpc-3*, which results in loss of eIF2α phosphorylation, preferentially increased the abundances of large proteins encoded by mRNAs with non-optimal codon usage (Figure 4A and B). This result suggests that a high local concentration of phosphorylated eIF2α specifically inhibits translation initiation of long CDS mRNAs with non-optimal codon usage. This notion is also consistent with our discovery that the correlation between codon usage and ribosome density increases as CDS length increases (Figure 2E). It is important to note that such an effect on ribosome density occurs despite of the known dramatic opposing effect of codon usage on ribosome occupancy because of its role in elongation rate in *Neurospora* (21,23), suggesting that codon usage feeds back on translation initiation in an mRNA-specific manner.

Consistent with our model that translation elongation feeds back to influence mRNA-specific translation initiation, it was previously proposed that translation initiation and elongation coordinate with each other to optimize protein production: mRNAs that encode high-abundance proteins usually have high translation initiation rates, fast elongation rates, and optimal codon usage (90). In addition, low levels of eIF2α phosphorylation may have specific rather than broad effects on translation (50,62). Importantly, it was previously shown that certain chemical modifications of mRNA transcribed *in vitro* can specifically enhance its translation in cells through attenuating eIF2α phosphorylation and increasing translation initiation (96). The specific effect of eIF2α phosphorylation can be caused by the compartmentalization of translation or the local ribosome recycling for translation re-initiation of circularized mRNAs (97–100).

Our data indicate that the presence of rare codons activates CPC-3 to phosphorylate eIF2α. The yeast and mammalian homolog GCN2 has been shown to be associated with ribosomes and such association is important for GCN2 activation (101–104). The interaction of GCN2 with the ribosomal P-stalk can potently activate GCN2 in the absence of uncharged tRNA (59,60). Therefore, it is likely that rare codons trigger ribosome pausing, which may promote the interaction between GCN2 (or CPC-3) and ribosomes, resulting in the kinase activation and the subsequent eIF2α phosphorylation. It was also previously shown that ribosomes have different conformations at different stages of the elongation cycle (84). It is possible that the ribosome conformation at a specific functional state with an empty A site promotes the interaction between ribosomes and CPC-3 and the latter activation.

As expected, deletion of *cpc-3* resulted in a general increase of protein synthesis, confirming the roles of eIF2α phosphorylation in inhibiting translation initiation. Unexpectedly, however, deletion of *cpc-3* in *Neurospora* also had a major impact on translation elongation rate, and this effect was also dependent on codon usage and CDS length. The increased translation elongation rate in the Δ*cpc*-3 strain was demonstrated by three independent methods: the relative codon decoding rates determined by ribosome profiling, the *in vitro* translation assay that was used to directly compare translation elongation rates, and the *in vivo* protein conformation reporter that is sensitive to elongation rate change. Although the elongation rates of all codons were increased in the Δ*cpc-3* strain, the effects were codon usage-dependent: Deletion of *cpc-3* preferentially increased elongation rates of mRNAs rich in rare codons, indicating that CPC-3 regulates elongation rates in a codon usage-dependent manner. Thus, in the WT strain, CPC-3 amplifies the codon usage effect on elongation speed so that codon decoding rates for optimal codons are much faster than those for rare codons.

The negative correlations between CDS length and protein abundance, translation initiation rate, and ribosome density suggest that increasing ORF length may decrease the translation initiation efficiency (22,36–40). Our results here suggest that both CPC-3-dependent and CPC-3-independent mechanisms are involved in the CDS length-dependent regulation on protein production. The CPC-3-independent mechanism may be due to the less efficient mRNA circularization, ribosome re-initiation or ribosome recycling for long CDS mRNAs than for short CDS mRNAs (39,41). Because protein production rate should be mostly determined by translation initiation rate on mRNAs unless there are significant amounts of translation abortion events (40), the up-regulated protein synthesis rates (including the 3 × WT EDP reporter) in the Δ*cpc-3* strain suggest their increased translation initiation. However, if there are strong ribosome stalling or premature termination during translation elongation, the increase of the translation elongation can also promote translation efficiency. Our results showed that CPC-3 plays an important role in regulating translation elongation in addition to its role in regulating translation initiation. Therefore, for the feedback mechanism mediated by codon usage and CDS length, the effects of CPC-3 and eIF2α phosphorylation on translation initiation should play an important role in regulating protein synthesis levels. In addition, the role of CPC-3 in translation elongation can also contribute to translation efficiency by regulating ribosome stalling or premature translation termination events (21,23).

Codon decoding rates are also regulated by CDS length: the rates are slower for mRNAs with long CDS regions and faster for mRNAs with short CDS regions. This phenomenon was observed in both *N. crassa* and *S. cerevisiae*, suggesting a conserved mechanism regulating translation elongation speed in eukaryotes. Because the elongation rate regulates co-translation folding process, and large proteins have more structural domains and should be more prone to be misfolded than small proteins (93,105–107), a slow elongation rate likely promotes optimal co-translational folding of large proteins.

In higher eukaryotes, in addition to GCN2, the eIF2α phosphorylation at Ser 51 can also be mediated by protein kinase R, PKR-like endoplasmic reticulum kinase and heme-regulated inhibitor (42,50,51). Therefore, it is possible that these additional kinases may also contribute to the feedback process from translation elongation to initiation. In addition, although our results demonstrated the involvement of CPC-3 in regulating the feedback mechanism from elongation to initiation in a codon usage and CDS length-dependent manner, CPC-3 independent mechanism may also exist.

Although how CPC-3 slows down elongation rate is not known, GCN2 in both yeast and mammalian cells has been shown to interact with the translation elongation factor eEF1A and this interaction keeps GCN2 inactive under nutrient-replete conditions (108–110). It is possible that this interaction also negatively influences the ability of eEF1A to deliver cognate aminoacyl tRNAs to the ribosomal A‐site during elongation. Together, our results here suggest that translation elongation can feed back on both translation initiation and elongation kinetics through a mechanism that depends on codon usage and CDS length to allow optimal synthesis of proteins of different sizes.

## DATA AVAILABILITY

Ribosome profiling and RNA-seq data have been submitted to the NCBI Gene Expression Omnibus under accession number GSE168595. Customized scripts used for ribosome profiling analyses were deposited at https://github.com/lxlscc0715/scripts-for-ribosome-profiling-and-RNA-seq.

## Supplementary Material

gkab729_Supplemental_Files
