# Supplementary material for: Codon usage and protein length-dependent feedback from translation elongation regulates translation initiation and elongation speed
Source: Nucleic Acids Res. 2021 Aug 20;49(16):9404–23. doi: 10.1093/nar/gkab729 (PMC8450115; doi:10.1093/nar/gkab729)
Supplement: gkab729_Supplemental_Files [file gkab729_supplemental_files.zip › supplemental revised figures.pdf]

Supplemental Figures and Tables

Figure S1. Western blot analyses of the *cpc-3* deletion strain and its complementation strain. The eIF2α phosphorylation in the *cpc-3* deletion strain was completely abolished and can be rescued by the expression of Flag-tagged CPC-3 (under the control of *ccg-1* promoter). Comp-1 and -2 are the *cpc-3* deletion strains expressing Flag-CPC-3. The eIF2α protein level in each sample was used as protein loading control.

Figure S2. Sequence alignments of WT/OPT *edp* (A), *GFP* (B), and *Luc* (C) open reading frames. Nucleotides with blue background are conserved sites.

Figure S3. Correlation between CDS lengths and mRNA levels does not depend on codon usage. Corresponding to Figure 1. Line plots showing the correlation between CDS lengths and mRNA levels with tAIs increased. All the detected genes were ranked by their tAIs, and Pearson's correlation coefficient was calculated in each window which continuously slides from low to high tAIs. Each window contains 500 genes. mRNA levels are from RNA-seq data for *N. crassa*.

Figure S4. (A) Densitometric analyses of the relative EDP protein levels in the four indicated strains from three independent experiments. Replotted from the results in Figure 2B. (B) Comparison of the relative ribosome density levels among the indicated four reporter mRNAs.

Figure S5. Classification of genes based on their codon usage and CDS length. Corresponding to Figure 2F. All the genes in *N. crassa* genome were divided into three different groups based on their CBI values (as indicated) and each group contains 3405, 3616 and 2699 genes respectively. In each group, genes were ranked by their CDS lengths from short to long, and then were further divided into three bins based on their CDS lengths: the first and the third bin contain 1000 genes respectively. Genes in four bins (colored by green, red, blue and purple respectively) were selected for functional enrichment analysis and the results are shown in Figure 2F and Table S2.

Figure S6. Constructs used in cell-free *in vitro* translation system. (A, B) Upper panels: Graphical representation of the structures of the Luc mRNAs and 1 × /2 × GFP mRNAs. For all the GFP mRNAs, codons for 3 × Flag at N-terminus are the same. For all the mRNAs in (A) and (B), polyA lengths are the same. Lower panels: SDS-PAGE gel analysis showing the ^{35}S-Met labeled translational products from WT/OPT Luc mRNAs in micrococcal nuclease treated *Neurospora* cell-free lysates.

Figure S7. Densitometric analyses of the western blot results of the EDP levels in the four indiated strains from three independent experiments. The EDP protein level in the 1xWT strain was set as 1.0. Replotted from the same results as in Figure 4B.

Figure S8. **Polysome profiling analyses of the WT strain and the Δ*cpc-3* strain. (A)** Comparison of the polysome profiles between the WT and Δ*cpc-3* strains. The positions corresponding to the 40S and 60S subunits, the 80S monosomes, and polysomal ribosomes are indicated. Recorded relative arbitrary units (A.U.) were plotted. **(B)** Quantification of the polysome profiles in (A). The ratios between polysomes and monosome fractions are indicated for the WT and Δ*cpc-3* strains. Data are means with SD (n = 3). * $P < 0.05$; as determined by Student's two-tailed t test.

Figure S9. Validation of the phosphorylation of the WT-EDP reporter. The hyper-phosphorylated forms of the WT-EDP in the Δ *cpc-3* strain disappeared when the protein samples were treated with Lambda Protein Phosphatase (Lambda PP). PP inhibitors: phosphatase inhibitors added into the protein extraction buffer.

Figure S10. Deletion of *cpc-3* preferentially increases the protein abundance of large proteins. **(A)** The individual protein levels determined by quantitative MS (TMT labeling) analysis in the Δ *cpc-3* strain were compared to those in the Δ *cpc-3* strain expressing the Flag-tagged CPC-3. Proteins were ranked by their lengths from short to long. The proportions of genes with significantly increased protein levels in the Δ *cpc-3* strain increase in scanning 1000 gene-windows as the protein sizes increase. The cut-off of differentially expressed protein levels

is FDR < 0.05. Data are means with SD (n = 4). Details see Table S3. **(B)** Comparison of the protein length profiles of the predicted *N. crassa* proteome, all detected proteins by the MS analysis and the up-regulated proteins in the Δ *cpc-3* strain. Boxplot showing that the up-regulated proteins in the Δ*cpc-3* strain were preferentially larger proteins comparing to the proteome average or the average of total detected proteins by the MS. P-values were shown, as determined by Welch t-test.

Figure S11. Paired comparison of RCDTs of all codons in mRNAs of long and short CDS regions in the WT and Δ*cpc-3* strains. Related to Figure 7C-E. Comparison of the RCDTs of all codons in the mRNAs with CDS > 600 aa and those < 300 aa in the WT and Δ *cpc-3* strains. For each codon family, the codon usage frequency increases from the first codon to the last codon.

Table S1. The results of MS experiments quantifying the relative protein levels in the *N. crassa* WT strain. Related to Figure 1.

Table S2. Gene functional enrichment analyses based codon usage and CDS length. Related to Figure 2F and Figure S4.

Table S3. The results of the quantitative MS (TMT labeling) analysis for the Δ *cpc-3* strain and the Δ *cpc-3* complementation strain expressing the Flag-tagged CPC-3 (control).

Figure S1

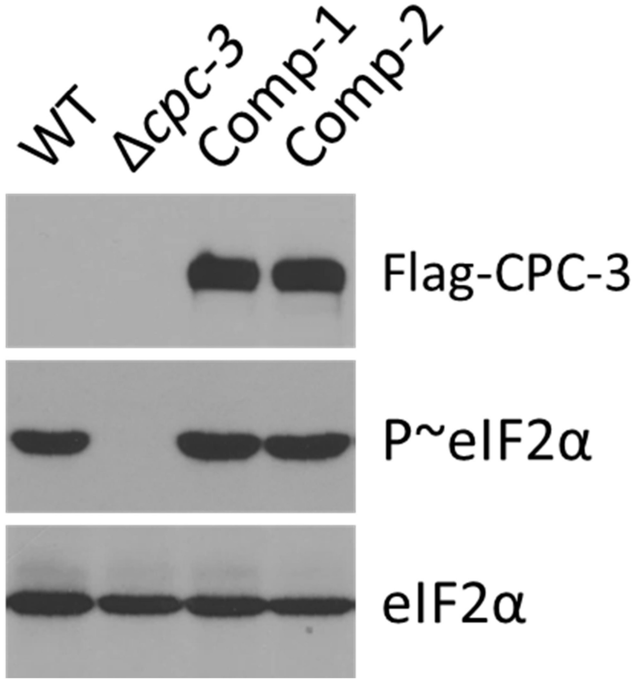

Figure S2

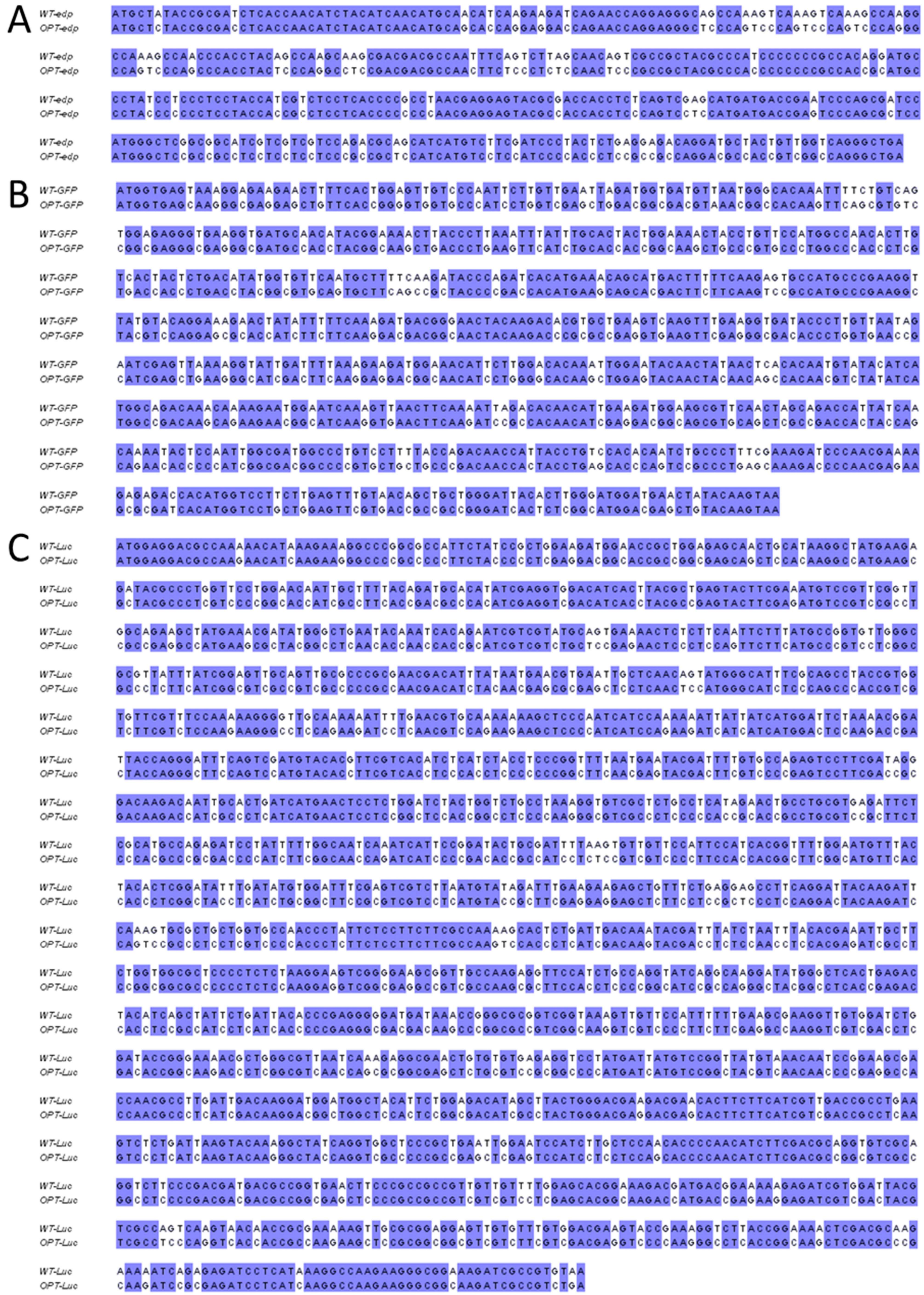

Figure S3

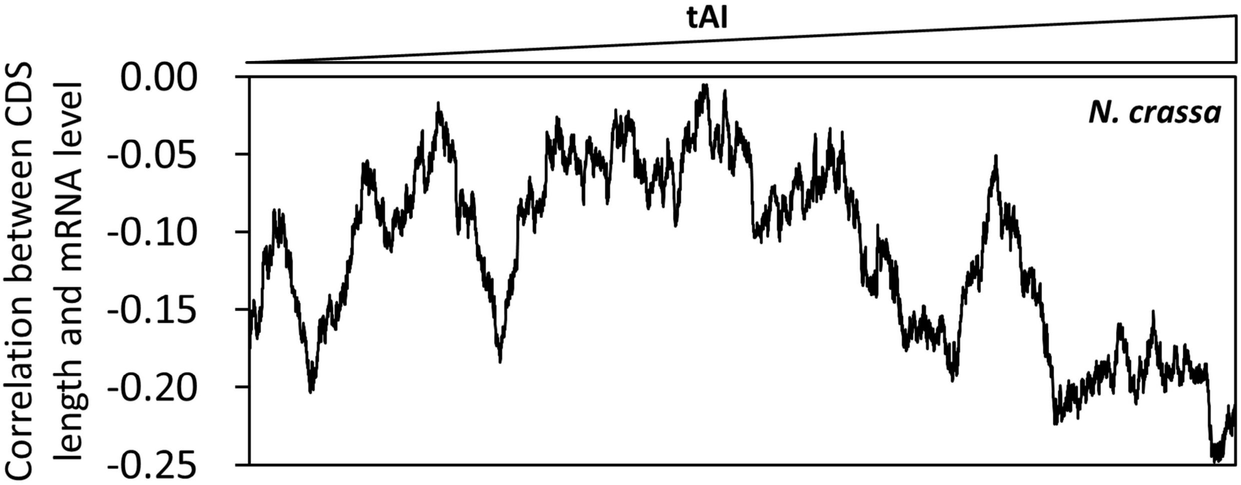

tAI
N. crassa
Correlation between CDS length and mRNA level
0.00
-0.05
-0.10
-0.15
-0.20
-0.25

Figure S4

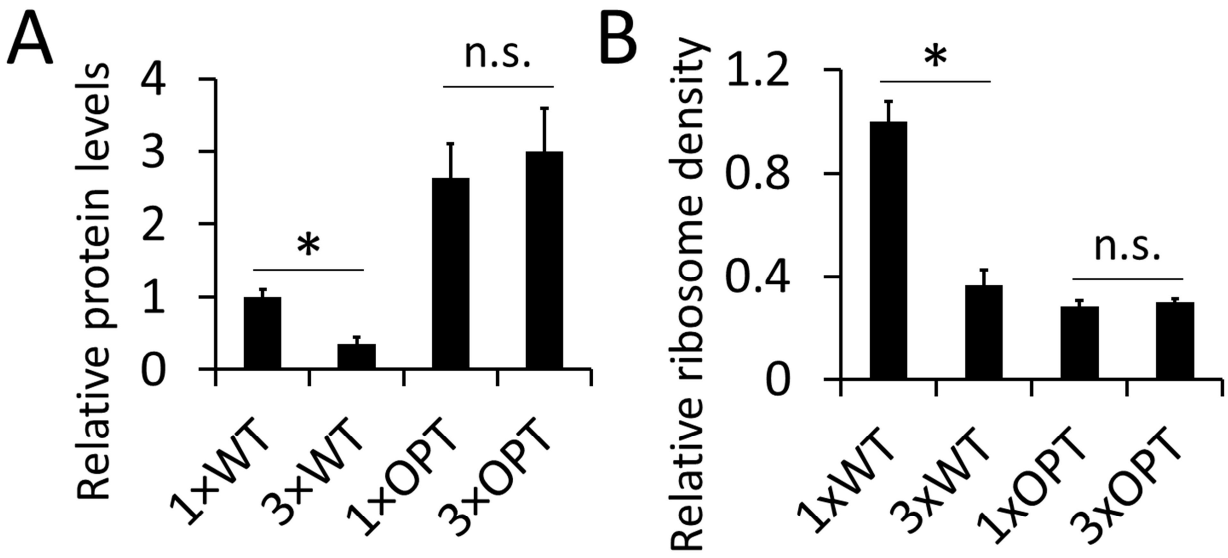
A
Relative protein levels
4
3
2
1
0
*
n.s.
1×WT
3×WT
1×OPT
3×OPT
B
Relative ribosome density
1.2
0.8
0.4
0
*
n.s.
1xWT
3xWT
1xOPT
3xOPT

Figure S5

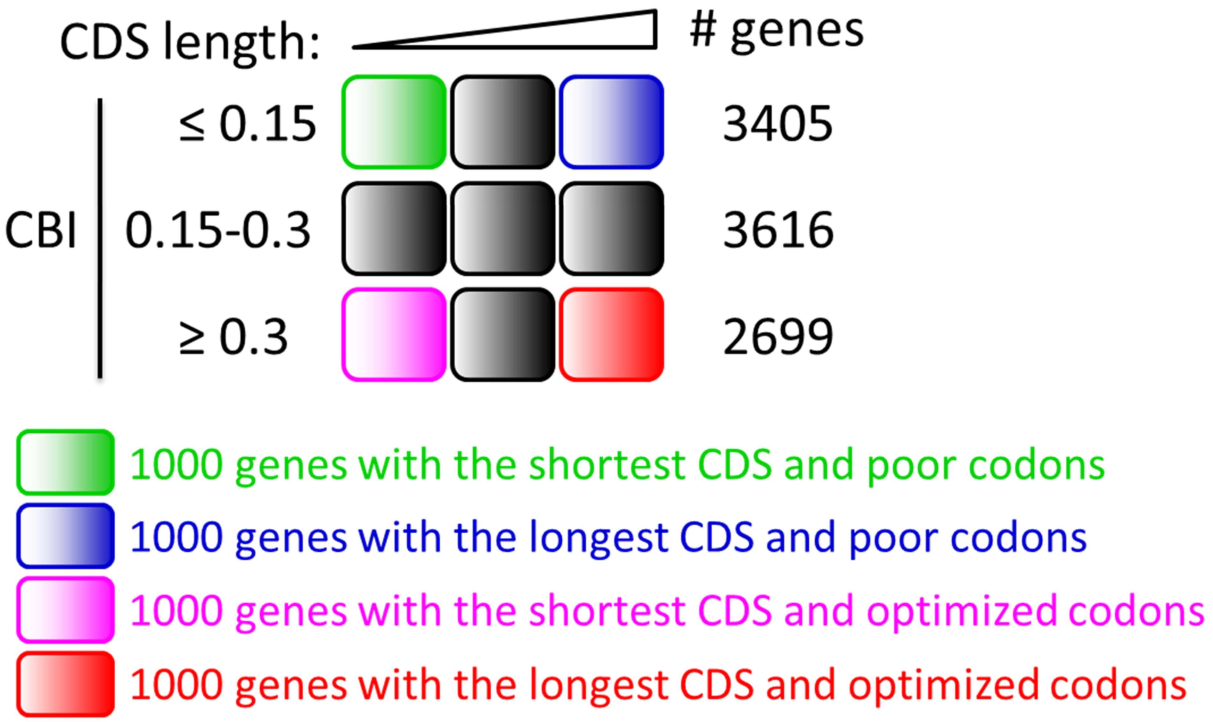
CDS length:
genes
CBI
≤ 0.15
0.15-0.3
≥ 0.3
3405
3616
2699
1000 genes with the shortest CDS and poor codons
1000 genes with the longest CDS and poor codons
1000 genes with the shortest CDS and optimized codons
1000 genes with the longest CDS and optimized codons

Figure S6

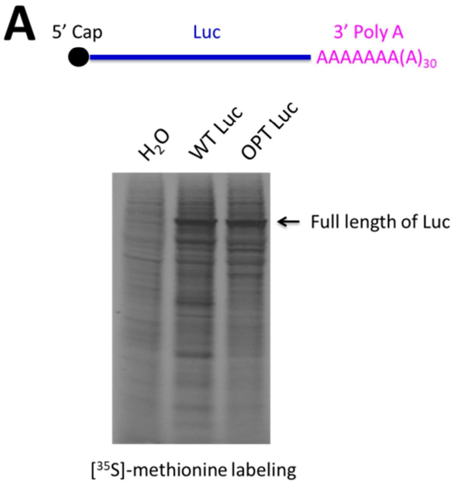
A
5' Cap
Luc
3' Poly A
AAAAAAA(A)30
H2O
WT Luc
OPT Luc
Full length of Luc
[35S]-methionine labeling

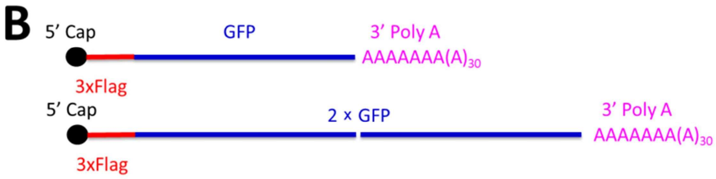
B
5' Cap
GFP
3' Poly A
AAAAAAA(A)30
3xFlag
5' Cap
2 × GFP
3' Poly A
AAAAAAA(A)30
3xFlag

Figure S7

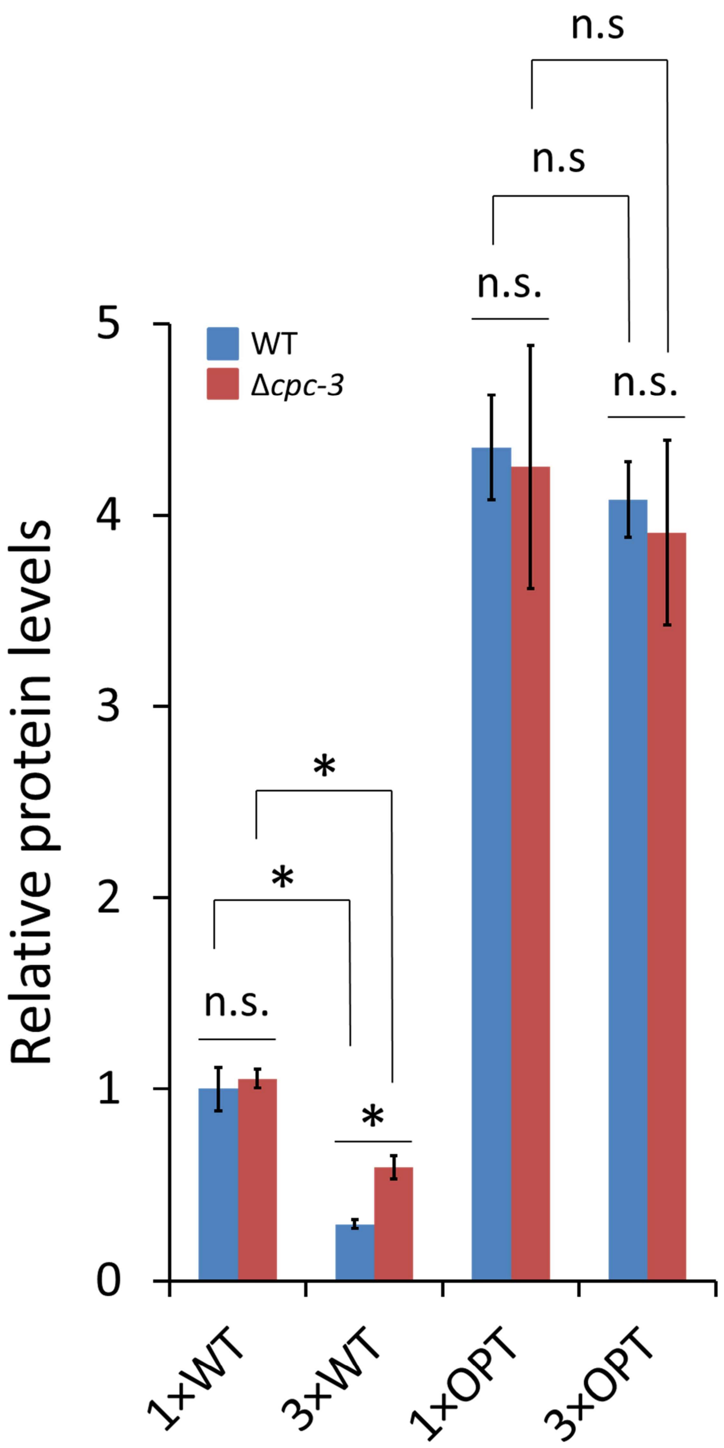

n.s
n.s
n.s.
n.s.
5
4
3
2
1
0
WT
Δcpc-3
Relative protein levels
*
*
n.s.
*
1×WT
3×WT
1×OPT
3×OPT

Figure S8

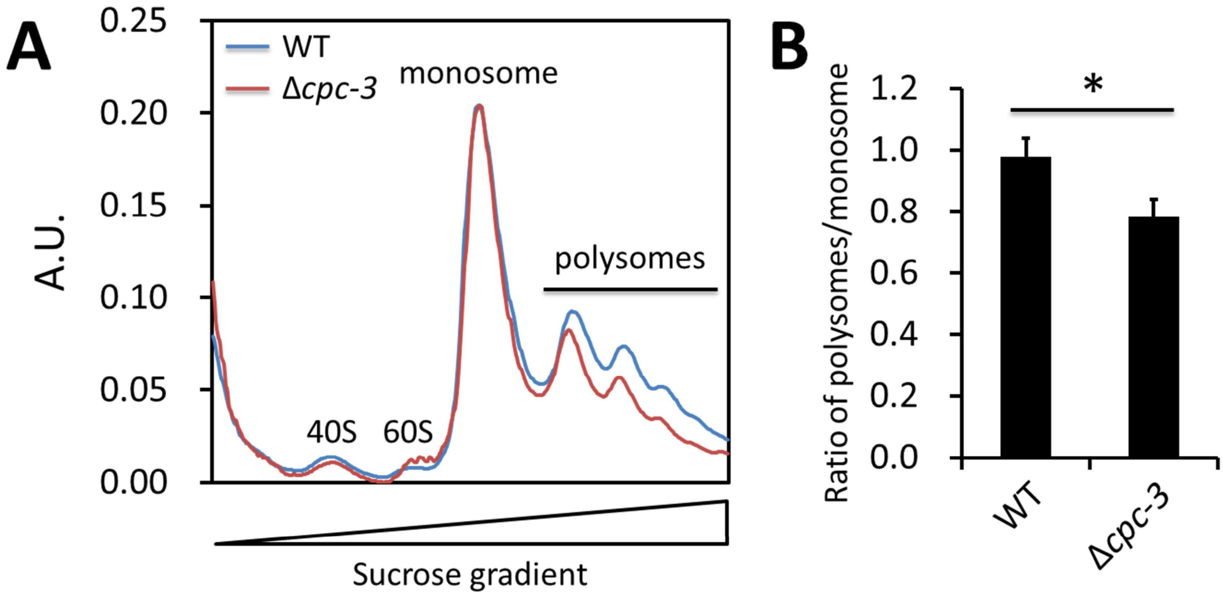
A
0.25
0.20
0.15
0.10
0.05
0.00
A.U.
WT
Δcpc-3
monosome
40S
60S
polysomes
Sucrose gradient
B
Ratio of polysomes/monosome
1.2
1.0
0.8
0.6
0.4
0.2
0.0
*
WT
Δcpc-3

Figure S9

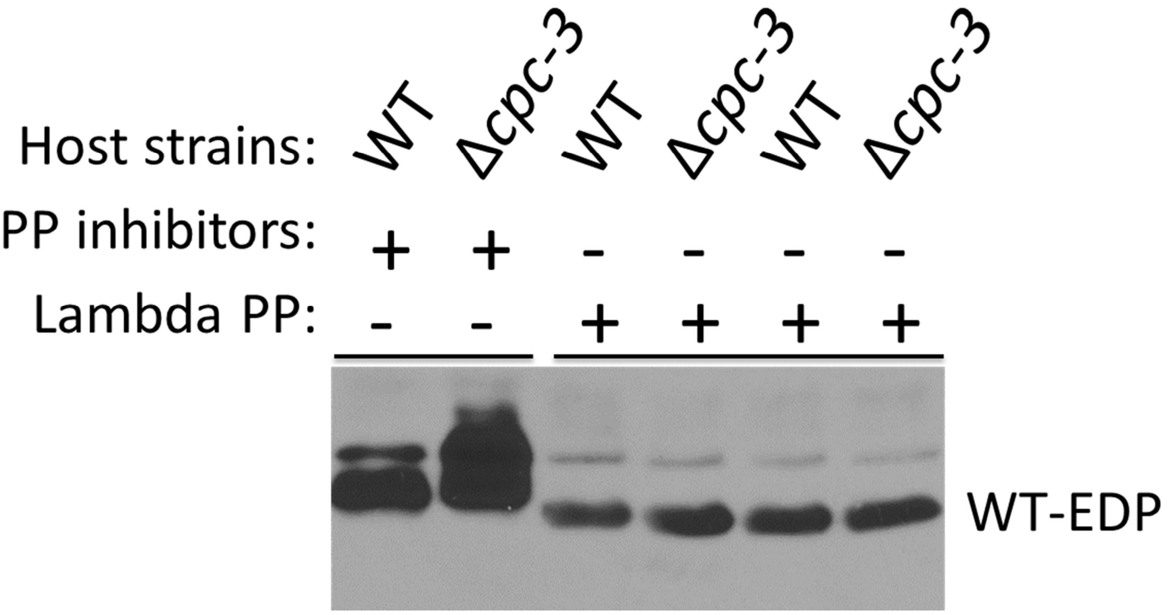
Host strains:
WT
Δcpc-3
WT
Δcpc-3
WT
Δcpc-3
PP inhibitors:
+ + - - - -
Lambda PP:
- - + + + +
WT-EDP

Figure S10

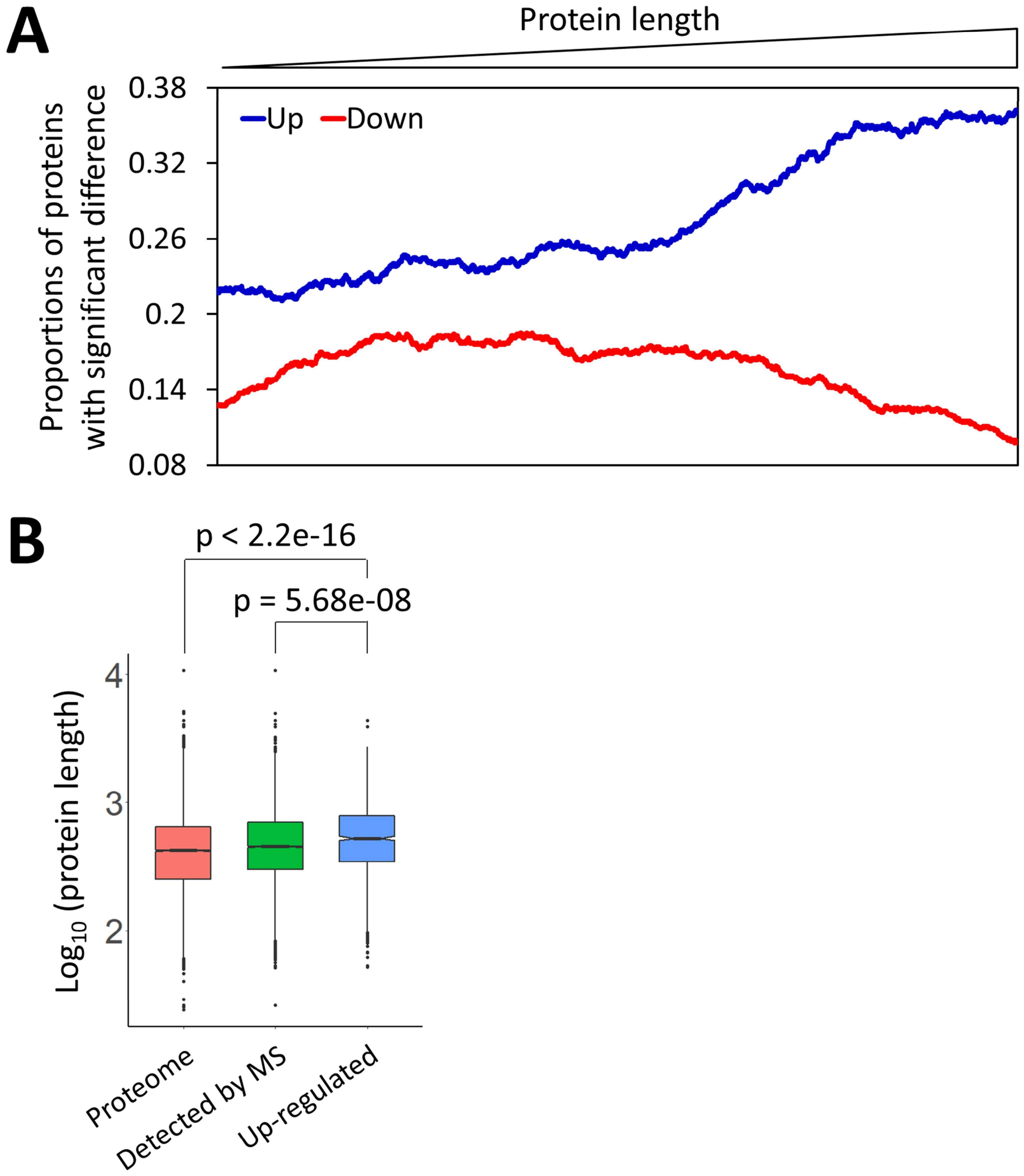
A
Protein length
Up
Down
Proportions of proteins with significant difference
0.38
0.32
0.26
0.2
0.14
0.08
B
p < 2.2e-16
p = 5.68e-08
Log10 (protein length)
4
3
2
Proteome
Detected by MS
Up-regulated

Figure S11

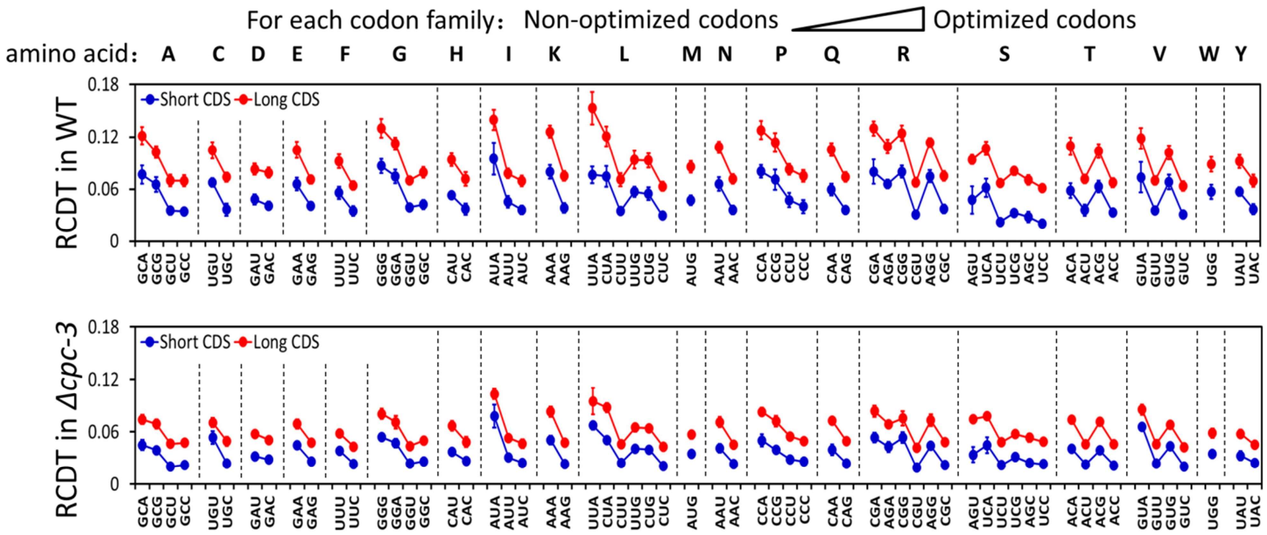
For each codon family: Non-optimized codons Optimized codons
amino acid: A C D E F G H I K L M N P Q R S T V W Y
Short CDS
Long CDS
RCDT in WT
RCDT in Δcpc-3
0.18
0.12
0.06
0
